# A ‘wiring diagram’ for sink strength traits impacting wheat yield potential

**DOI:** 10.1093/jxb/erac410

**Published:** 2022-11-05

**Authors:** Gustavo A Slafer, M John Foulkes, Matthew P Reynolds, Erik H Murchie, Elizabete Carmo-Silva, Richard Flavell, Jeff Gwyn, Mark Sawkins, Simon Griffiths

**Affiliations:** Department of Crop and Forest Sciences, University of Lleida–AGROTECNIO-CERCA Center, Av. R. Roure 191, 25198 Lleida, Spain; ICREA (Catalonian Institution for Research and Advanced Studies), Barcelona, Spain; Plant and Crop Sciences, School of Biosciences, University of Nottingham, Leicestershire LE12 5RD, UK; International Maize and Wheat Improvement Center (CIMMYT), Km. 45, Carretera Mexico, El Batan, Texcoco, Mexico; Plant and Crop Sciences, School of Biosciences, University of Nottingham, Leicestershire LE12 5RD, UK; Lancaster Environment Centre, Lancaster University, Lancaster LA1 4YQ, UK; International Wheat Yield Partnership, 1500 Research Parkway, College Station, TX 77843, USA; International Wheat Yield Partnership, 1500 Research Parkway, College Station, TX 77843, USA; International Wheat Yield Partnership, 1500 Research Parkway, College Station, TX 77843, USA; John Innes Centre, Norwich Research Park, Colney Ln, Norwich NR4 7UH, UK; MPI of Molecular Plant Physiology, Germany

**Keywords:** Breeding, grain number, grain weight, harvest index, source–sink, yield components, yield physiology

## Abstract

Identifying traits for improving sink strength is a bottleneck to increasing wheat yield. The interacting processes determining sink strength and yield potential are reviewed and visualized in a set of ‘wiring diagrams’, covering critical phases of development (and summarizing known underlying genetics). Using this framework, we reviewed and assembled the main traits determining sink strength and identified research gaps and potential hypotheses to be tested for achieving gains in sink strength. In pre-anthesis, grain number could be increased through: (i) enhanced spike growth associated with optimized floret development and/or a reduction in specific stem–internode lengths and (ii) improved fruiting efficiency through an accelerated rate of floret development, improved partitioning between spikes, or optimized spike cytokinin levels. In post-anthesis, grain, sink strength could be augmented through manipulation of grain size potential via ovary size and/or endosperm cell division and expansion. Prospects for improving spike vascular architecture to support all rapidly growing florets, enabling the improved flow of assimilate, are also discussed. Finally, we considered the prospects for enhancing grain weight realization in relation to genetic variation in stay-green traits as well as stem carbohydrate remobilization. The wiring diagrams provide a potential workspace for breeders and crop scientists to achieve yield gains in wheat and other field crops.

## Teaser

Using a set of ‘wiring diagrams’ in critical pre- and post-anthesis periods, we reviewed traits determining wheat sink strength, relevant for further improving yield potential, identifying research gaps to be studied.

## Introduction

**Fig. 1.**
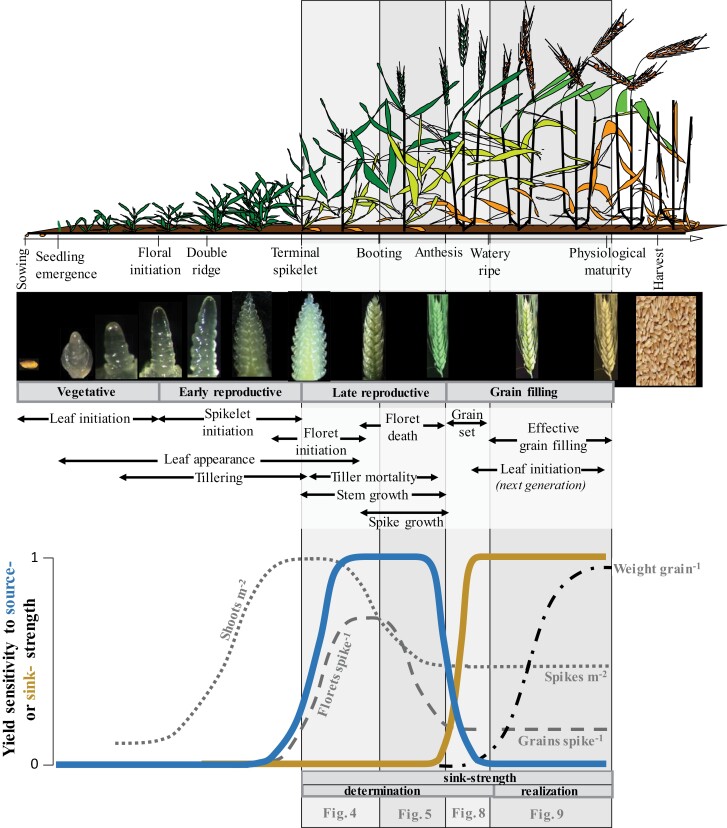
Graphical time course of wheat phenology indicating key developmental stages (organs are not at the same scale and the relative duration of different phases varies depending on genotypes and environments), illustrating the appearance of the apex/spike, the timing of differentiation and growth of organs, and the degree of source or sink limitation for yield [from terminal spikelet stage to shortly after anthesis, source strength determines the grain sink strength (number of grains and their potential size), that then limits the potential accumulation of yield during the effective grain-filling period]. That is, pre-anthesis source strength influences yield because it determines sink strength during grain filling. This defines periods over which sink strength forms (in this study divided into three dedicated WDs, later shown in Figs 4, 5 and 8) and realized (during the effective period of grain filling, with a WD shown in Fig. 9) (see shaded boxes behind the schemes). Based on an original scheme from Slafer and Rawson (1994); and from Ochagavía *et al.* (2021) and Slafer *et al.* (2021). Reprinted with permission from Elsevier.

The concept of a physiological sink refers to organs which are net ‘receivers’ of photosynthate and other metabolites that are then either consumed by the organ for its own metabolism or stored (to be used later by other sinks). Identifying appropriate traits responsible for improving sink strength is one of the current bottlenecks to further increases in yield potential (Reynolds *et al.*, 2021). In this context, we mostly limit the concept to the reproductive sinks, and consider sink strength as the collective capacity of grains to accumulate dry matter as defined by the number of grains set per unit area and their potential weight. Although this review is sink oriented, it is necessary to appreciate the crop’s photoassimilate supply (source strength, considered specifically in the companion paper; Murchie *et al.*, 2023), which provides energy and molecules for constructing sinks, and also the interactions between source and sink. Briefly (for more detailed consideration, please see the discussion offered in Slafer *et al.*, 2021; Reynolds *et al.*, 2022), although yield is being formed throughout the whole crop cycle (Slafer and Rawson, 1994), the different phases have more or less relevance, depending on both their sensitivity to environmental factors and the consequences of that sensitivity on yield. Most frequently, yield is limited by sink strength during the effective period of grain filling, and source limited from the terminal spikelet initiation up to the onset of grain filling (Fig. 1). For instance, in a recent study by Dreccer *et al.* (2022) subjecting wheat plants to changes in resources (CO_2_) and signals (red:far red ratio) during different phases, it was demonstrated that yield responses were concentrated around treatments applied when sink strength is built up, while changes during grain filling were far less effective (as sink strength has already been set and there is no source limitation to filling the grains). This reinforces, with a new approach, what has been the most frequent scenario in the literature (e.g. see reviews by Slafer, 2003; Fischer, 2011; Foulkes *et al.*, 2011; Reynolds *et al.*, 2021, 2022; and a wealth of references quoted therein), and extends the knowledge on the effects of resources on signals, in agreement with what was found when wheat was subject to the contrasting effects of poorer light quality (Ugarte *et al.*, 2010) and longer photoperiods (Gonzalez *et al.*, 2003, 2005; Ferrante *et al.*, 2020).

During the period from terminal spikelet to slightly after anthesis, the number of grains per unit land area is set mainly through tiller mortality, floret primordia death, and grain abortion from anthesis to the watery ripe grain stage; potential grain size is established from booting to the watery ripe stage (Fig. 1; and see also Slafer *et al.*, 2021). Crop growth before the terminal spikelet is less critical for yield determination provided crop growth is maximized from the terminal spikelet onwards (an empiric proof of concept for this is that plant densities optimizing yield potential are far lower than those maximizing biomass in early stages).

The interacting processes that determine yield potential are complex (Fig. 1). To help visualize and analyse them, an interactive graphical representation—or set of ‘wiring diagrams’ (WDs), covering the most critical phases of crop development—has been developed (Reynolds *et al.*, 2022). The present review provides additional WDs to aid understanding and further analysis in much greater detail. We focus here on traits and relationships that are relevant for sink strength determination and have divided them into four phenological phases, with a dedicated WD for each of them, delimited by the stages of onset of stem elongation (roughly coinciding with terminal spikelet), booting, anthesis, watery ripe, and physiological maturity (Fig. 1). In the first three phases, different processes that are responsible for the determination of the sink strength are considered, while in the last phase the factors defining whether the crop realizes the sink strength-determined yield are described (Fig. 1). A companion review (Murchie *et al.*, 2023) presents WDs for stages of crop development responsible for source strength. Appreciation of the source–sink interactions outlined in both reviews is essential to understand the determination of grain yield because the separation of the full set of WD reviews into source and sink papers, primarily for the convenience of publication, can easily minimize the importance of the interactions between tissues and organs that occur at different times in crop development.

**Fig. 2.**
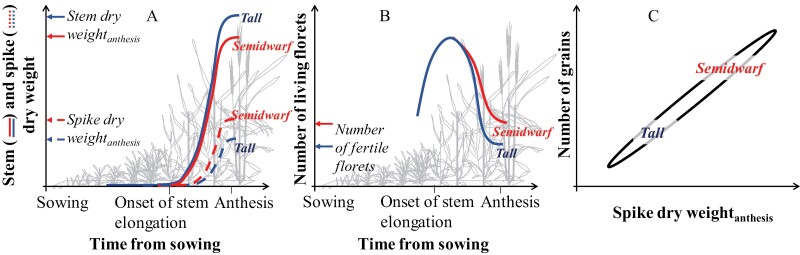
Schematic representation of how the introgression of semi-dwarfing alleles increased sink strength in wheat. The *Rht-B1* and *Rht-D1* genes (formerly known as *Rht-1* and *Rht-2*) restricted the capacity of the stem to grow, allowing more assimilates to be allocated to the juvenile spike before anthesis (A) (Fischer and Stockman, 1986; Siddique *et al.*, 1989; Slafer and Andrade, 1993; Miralles *et al.*, 1998). Floret development takes place in those juvenile spikes: many floret primordia are initiated and develop until a shortage of assimilates causes later initiated and weaker florets to die (e.g. Ferrante *et al.*, 2013a; Dreccer *et al.*, 2014). As semi-dwarf genotypes allocate more resources to the juvenile spike, more florets continue developing, thus reducing the rate of floret mortality and therefore increasing spike fertility (B) (e.g. Miralles *et al.*, 1998). This is the mechanistic basis for the well-reported linear relationship between the number of grains and the spike dry weight at anthesis (C), and explains how semi-dwarf alleles of *Rht-1* genes increased sink strength during grain filling and then increased the partitioning of aboveground biomass to yield, thereby resulting in a higher harvest index. This is a good empiric proof of concept of the model schematically represented in Fig. 1, where alleviation of restricted source strength for spike growth before anthesis increased yield through raising the level of sink strength.

The ratio between aboveground source and sink organs at harvest is described as the harvest index (HI; grain dry matter/aboveground dry matter). A step change in HI underpinned the dramatic yield gains of the Green Revolution, even though biomass was not improved and thereby necessarily setting an upper limit on yield. [Although the introgression of semi-dwarfing alleles (which is the foundation of the Green Revolution) did not increase biomass, the increased tolerance to lodging allowed higher levels of N fertilization, which did indeed increase biomass noticeably, but through the adapted management, not through crop genetics.] Semi-dwarf wheats had higher HI than their tall counterparts by creating a higher reproductive sink strength due to a shift in the proportion of dry matter from vegetative stem tissue to juvenile spikes in the pre-anthesis phase. This shift stimulates more floret primordia to continue developing normally and become fully developed, presumably fertile, florets (Fig. 2) able to set grains, thereby illustrating how source–sink interactions determine yield potential in wheat.

Steady genetic gains in HI were associated with yield improvement in wheat until the late 1990s (Reynolds *et al.*, 1999), and explained the success of wheat breeding across many countries with contrasting growing conditions (e.g. Austin *et al.*, 1980; Siddique *et al.*, 1989; Calderini *et al.*, 1995; Shearman *et al.*, 2005; Acreche *et al.*, 2008; Sadras and Lawson, 2011; del Pozo *et al.*, 2014; Flohr *et al.*, 2018; Lo Valvo *et al.*, 2018; Maeoka *et al.*, 2020; Mondal *et al.*, 2020). HI still shows significant genetic variation in modern wheat cultivars (Aisawi *et al.*, 2015), indicating that breeders have not yet fixed this trait.

The genetic basis of assimilate partitioning among different plant organs is only partially understood, and shows significant interactions between environment and genetic background (Griffiths *et al.*, 2015; Slafer *et al.*, 2015; Ferrante *et al.*, 2017). Nonetheless, in recent studies of wheat yield potential, large genetic ranges for dry matter partitioning among plant organs have been reported and some promising leads have been identified (Slafer *et al.*, 2015; Rivera-Amado *et al.*, 2019). This variation represents significant untapped yield potential, especially given the generally negative association between HI and biomass seen in most sets of modern cultivars (Aisawi *et al.*, 2015; Rivera-Amado *et al.*, 2019; Sierra-Gonzalez *et al.*, 2021). Through specific understanding of genetic and physiological mechanisms of increased HI, it should be possible to minimize the extent of this trade-off (Foulkes *et al.*, 201l).

In the WDs in this review, as in the companion paper focusing on source strength (Murchie *et al.*, 2023), numbered links have been developed for each of the specific ‘wires’ of the diagrams that correspond to different processes underlying sink strength. We have also commented on the genetic bases of these processes, when the established genetic bases were sufficiently soundly defined. The diagrams and evidence supporting them are necessarily ‘high level’ for the sake of clarity. Only in recent years have gene expression profiles and subcellular assays enriched knowledge of wheat traits. This review does not include these details, except in a few pertinent cases, because the aim has been to present a whole-crop picture.

To locate the focus in crop development of each section of text, the reader is referred to the chronological series of WDs (Figs 4, 5, 8, 9, which correspond to the phases indicated in Fig. 1). Each of the numbered wires in the figures is explained in the text. Some wires are active and important in more than one of the phases considered. In those cases, the description of that wire is provided only in the first phase where it is mentioned.

**Fig. 3.**
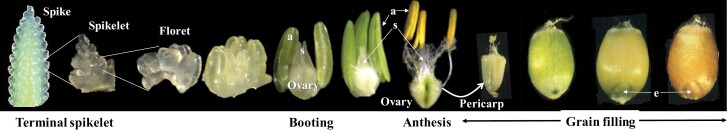
Changes in floret development from terminal spikelet to anthesis and in grain development from then to maturity (images not to scale). The ovary that becomes the pericarp is indicated. For reference, the anthers (a), stigma (s), and embryo (e) are also indicated. Reprinted from Ochagavía *et al.*, 2021, Copyright (2021), with permission from Elsevier.

**Fig. 4.**
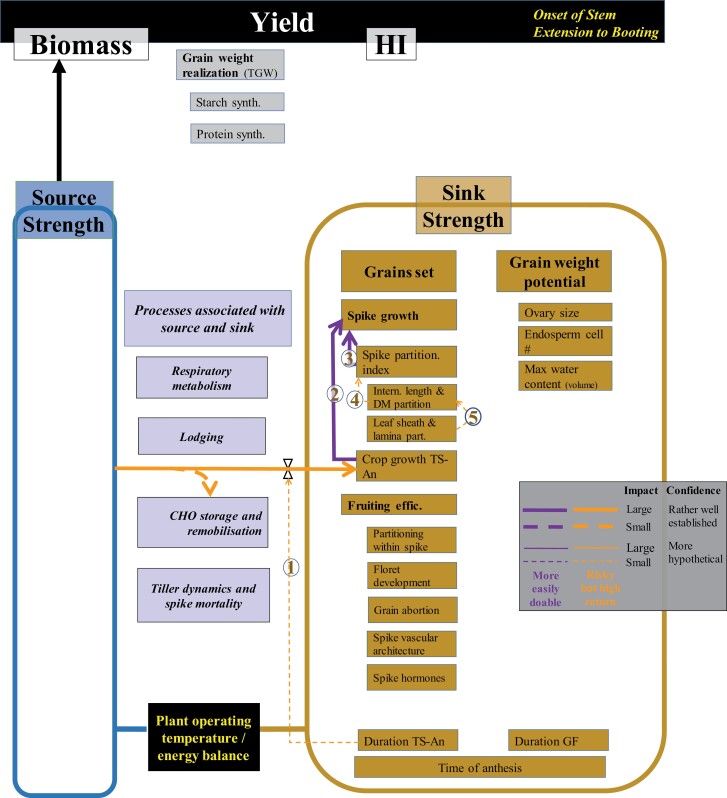
WD for sink strength determination in the period from the onset of stem elongation to booting. The thickness of the wires reflects the extent of the evidence on the link represented by the wire, the shape of the wire stands for the expected magnitude of impact on yield potential, and the colour of the wire reflects the ease/difficulty of exploiting the trait considered (see inset). Numbers on each wire refer to the links as cited and developed in the main text. TS, An, GF, DM, CHO, and TGW stand for terminal spikelet, anthesis, grain filling, dry matter, carbohydrates, and thousand grain weight, respectively.

**Fig. 5.**
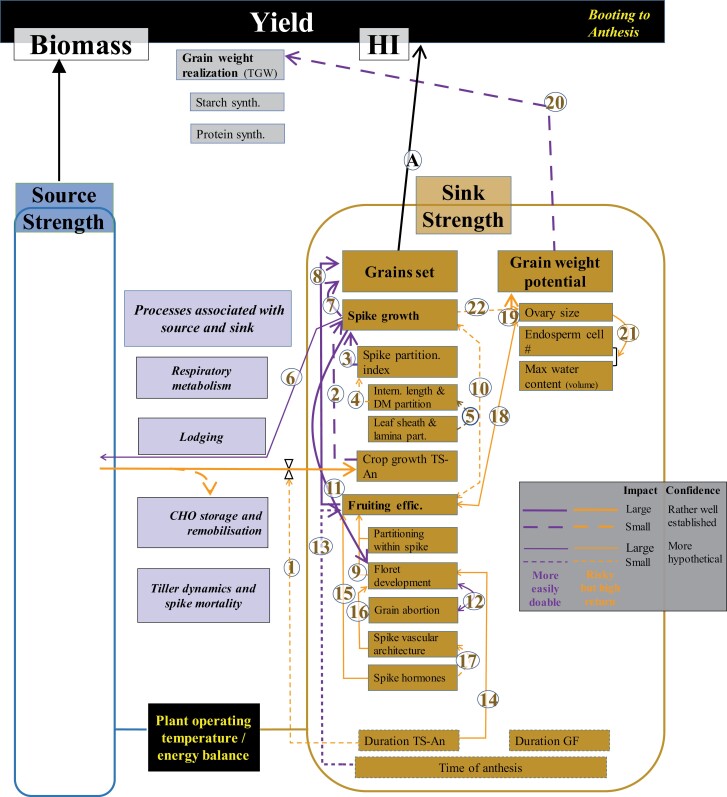
WD for sink strength determination in the period from booting to anthesis. Thickness, shape, and colour of the wires (inset) and numbers associated with them as described in Fig. 4. TS, An, GF, DM, CHO, and TGW stand for terminal spikelet, anthesis, grain filling, dry matter, carbohydrates, and thousand grain weight, respectively.

**Fig. 6.**
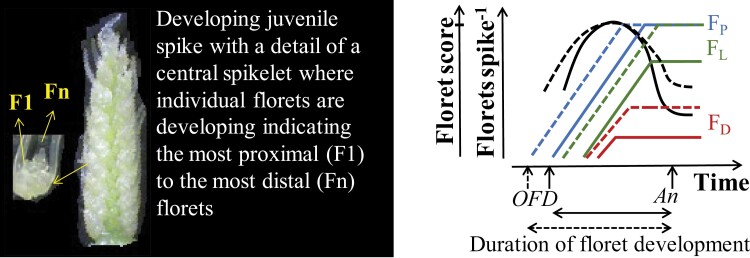
Dynamics of development of individual florets within spikelets, considering proximal florets (say the 2–3 florets most proximal to the rachis; FP), intermediate florets that are labile florets, depend on the G×E conditions, and may progress to produce a fertile floret or die (say florets 3–5, depending on the specific spikelet; FL), and distal florets (say florets 5–6 or more distal, that never produce fertile florets; FD). The scheme illustrates that when the period of floret development, from the onset of floret development (OFD) to anthesis (An) is extended (dashed lines), the longer period available for each floret to develop allows some labile florets to keep developing normally and to produce fertile florets instead of dying. Curved black lines represent the dynamics of the number of living floret primordia. The mark on the axis of floret score indicates when a floret primordium reaches the stage of fertile floret.

**Fig. 7.**
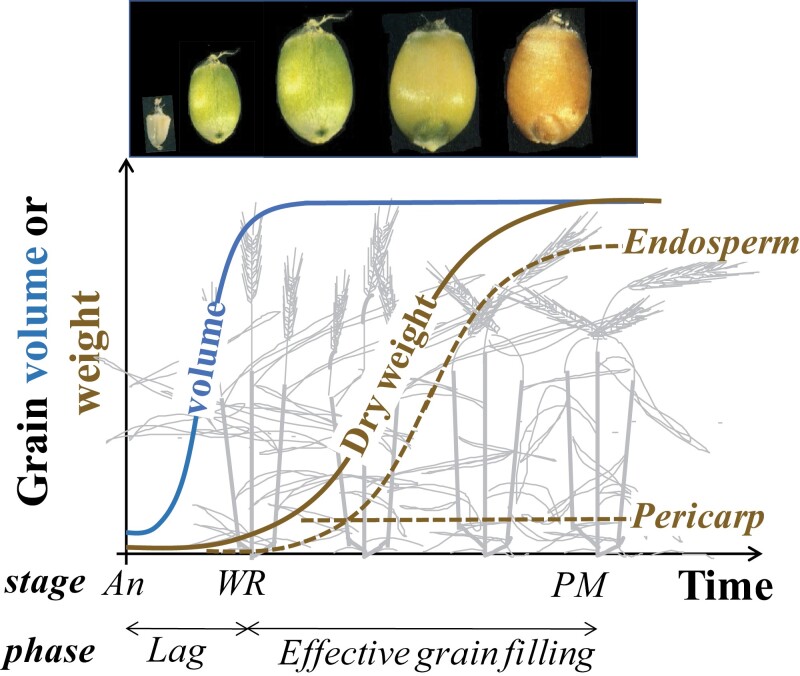
Schematic representation of the dynamics of grain volume and weight from anthesis to maturity (weight of pericarp and endosperm are shown in dotted lines). The stages of anthesis (An), watery ripe grain (WR), and physiological maturity (PM) as well as the duration of the lag phase and the effective grain-filling period are indicated underneath the abscissa. Changes in grain volume and colour from anthesis to maturity are illustrated on top. During the lag phase, non-aborting grains develop actively, producing the endosperm cells, and actively take up water which drives a large increase in volume. Grain volume is maximized first (establishing a likely upper threshold for grain size and, from then on, during the effective grain-filling period, assimilates are actively loaded into the grains and the actual grain weight is finally realized.

**Fig. 8.**
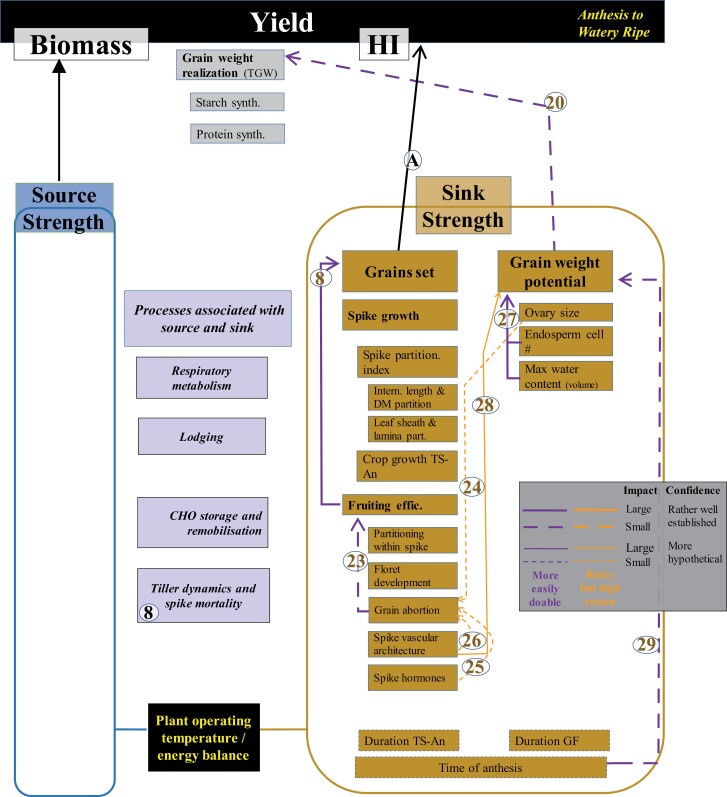
WD for sink strength determination in the period from anthesis to watery ripe. Thickness, shape, and colour of the wires (inset) and numbers associated with them as described in Fig. 4. TS, An, GF, DM, CHO, and TGW stand for terminal spikelet, anthesis, grain filling, dry matter, carbohydrates, and thousand grain weight, respectively.

**Fig. 9.**
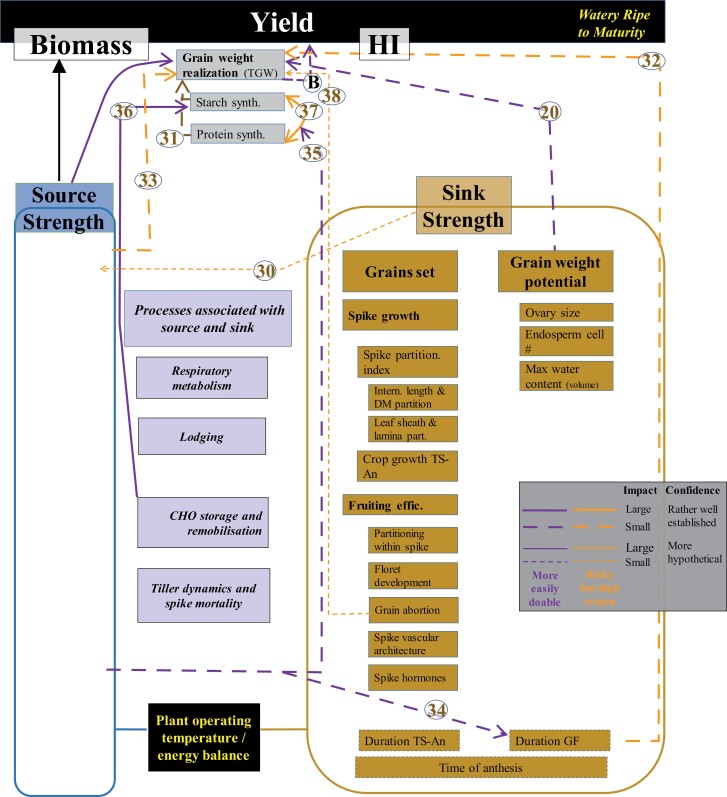
WD for sink strength determination in the period from watery ripe to physiological maturity. Thickness, shape, and colour of the wires (inset) and numbers associated with them as described in Fig. 4. TS, An, GF, DM, CHO, and TGW stand for terminal spikelet, anthesis, grain filling, dry matter, carbohydrates, and thousand grain weight, respectively.

## WDs from the onset of stem elongation to anthesis

The period of stem elongation, from terminal spikelet to anthesis (TS–An; embracing the growth of the juvenile spike in which florets are developing within spikelets), is critical for yield determination (Miralles *et al.*, 2000; Slafer *et al.*, 2001; Reynolds *et al.*, 2022). It is during this period that not only are the number of grains per m^2^ most strongly determined but also grain weight potential is, at least partly, set (Calderini *et al.*, 2001). Grain number is far more responsive to changes in crop growth and development during this phase than in any of the others (e.g. Slafer, 2003; Fischer, 2011, and references therein), in line with the idea that grain number is strongly influenced by photoassimilate supply before anthesis (Patrick and Colyvas, 2014; Reynolds *et al.*, 2022). Increasing the allocation of dry matter to juvenile spikes (considering the population of spikes, i.e. heavier individual spikes or more spikes becoming fertile) results in increases in grain number by preventing the death of some labile floret primordia (Ferrante *et al.*, 2013b, 2020; Dreccer *et al.*, 2014; Guo *et al.*, 2016). At the same time, the florets that continue to develop and become fertile actively grow, and the size attained by the carpels of these florets is an important component of the ultimate potential grain size after anthesis (Calderini *et al.*, 2021; and many references quoted therein), because the ovary wall of the florets becomes the pericarp of the grain (Fig. 3).

We divided the TS–An period into two WDs. The first focuses on the onset of stem elongation to booting (Fig. 4), when florets are being initiated but no significant biomass investment has been made in ovary growth; and the second from booting to anthesis (Fig. 5), when floret mortality takes place, and floral organs of the surviving florets grow noticeably, determining the final number of fertile florets and the final size of their ovaries.

### Grain set and determination of sink strength (major Link A, Figs 3, 4)

#### The physiology of grain set

In wheat, grain yield improvement has been highly associated with increased grain number per unit area (Canevara *et al.*, 1994; Slafer and Savin, 1994; Abbate *et al.*, 1995; Sayre *et al.*, 1997; Brancourt-Hulmel *et al.*, 2003; Shearman *et al.*, 2005; Miralles and Slafer, 2007; Peltonen-Sainio *et al.*, 2007). Current evidence suggests that sink strength during the grain-filling period (given by the number of grains and their potential size) remains a critical yield-limiting factor (Fischer, 1985, 2011; Beche *et al.*, 2014; LoValvo *et al.*, 2018). Grain number per unit area subsumes several components determined before anthesis: spikes per unit area, spikelets per spike, and grains per spikelet (see fig. 2 in Reynolds *et al.*, 2022). Since these components are subject to interaction with environmental conditions affecting tiller and/or floret production and survival and are determined by highly polygenic systems, grain number has a relatively low heritability (Sadras and Slafer, 2012). The period from onset of stem extension to anthesis is very important for the determination of grain number. It is generally accepted that the most critical stage covers the phase from late booting to anthesis (Slafer *et al.*, 1999). Indeed, experiments impairing crop growth at different stages of crop development have shown that affecting crop growth before the onset of stem elongation, at the time of spikelet initiation and tillering, does not affect grain number per m^2^, whilst doing so during the stem elongation phase (during which tiller mortality and floret development within spikelets occur) has a dramatic consequence on grains per m^2^ (Fischer, 1985, and a plethora of studies confirming the same in a range of countries, and genotypes; e.g. Savin and Slafer, 1991; Abbate *et al.*, 1995; Dreccer *et al.*, 2000; Demontes-Meynard and Jeuffroy, 2004; Ugarte *et al.*, 2007; Prasad and Djanaguiraman, 2014). Greater spike growth during this phase is strongly associated with higher grain number through increased floret survival (Calderini *et al.*, 1997; Fischer, 2007).

Grain growth of modern wheat cultivars is in general not strongly limited by the source [including actual canopy photosynthesis as well as remobilization of water-soluble carbohydrates (WSCs) during grain filling (Dreccer *et al.*, 1997; Borrás *et al.*, 2004; Calderini *et al.*, 2006)], although co-limitation by source may occur in some cases (Shearman *et al.*, 2005; Acreche and Slafer, 2009; Aisawi *et al.*, 2015) (see link B within the WDs for the phases from anthesis to maturity below). For example, photosynthesis during the post-anthesis period (at both leaf and canopy levels) seems to be responsive to increases in grain number, via source/sink manipulation treatments imposed around anthesis, as well as to genetic effects that increase grain number, even in modern cultivars with high grain numbers (e.g. Reynolds *et al.*, 2001, 2005; Acreche and Slafer, 2009).

#### The genetics of grain set

The purpose of the genetic narrative that follows is to highlight those studies which have identified the underlying genetic basis of grain number traits likely to deliver benefit to a modern breeding programme targeting high yield potential environments. Far fewer quantitative trait loci (QTLs) and marker trait associations (MTAs) are described for grains per unit area or its components than for grain size. This is not because there are fewer genes involved in the control of grain number. In fact, there are almost certainly more (Wang *et al.*, 2017), but many are of small genetic effect and subject to strong environmental interactions, and are therefore less likely to be detected in QTL analyses. They also result in low heritability (e.g. Sadras and Slafer, 2012).

Despite these challenges, there are good genetic routes that can complement phenotypic selection conducted by wheat breeders. For the complex reshuffling of small effect alleles, genomic selection shows great promise (Lozada *et al.*, 2019), while alleles with large effects are amenable to direct selection and positional cloning. There are few genes/QTLs that have been found to be robust and validated, ideally using near isogenic lines (NILs). These include—though are not limited to—*GNI-A1*, *Rht-B1* and *-D1*, and *TaAPO-A1* (Box 1; Table 1). The favourable alleles of these four genes are present at very high frequencies in elite wheat breeding pools. This is perhaps unsurprising as selection pressure for yield has been high, and with yield tightly linked to grain number, selection would have fixed these factors. There is unlikely, therefore, to be much scope for improved deployment of these alleles. However, the fact that the genes and causative polymorphisms are cloned opens up avenues to explore some of the molecular and biochemical pathways underlying grain number determination, many of which will be responsible for the relationships described below. These pathways are good targets for the study, manipulation, and deployment of induced and natural variation for increased grain number. In addition to the above-mentioned genes directly affecting grain number, phenology genes, mainly vernalization and photoperiod-sensitivity genes (*Vrn-1*, and *Ppd-1*, respectively), and a number of different genes acknowledged collectively as earliness *per se* genes (*Eps*), also ultimately affect grain number significantly (Box 2; Table 1).

**Box 1. Genetic effects with consistent effect on grain number and its components**

The following highlights three genetic effects found to control overall grain number per unit area and the components of grain number.

***GNI-1A*** on chromosome 2AL. Sakuma *et al.* (2018) identified a homeodomain leucine zipper class I (HD-Zip I) transcription factor, the expression of which was highest in the distal floret primordia of the spikelet and in parts of the rachilla. The authors demonstrated that the probable mode of action is to inhibit rachilla growth and development. In tetraploid wheat, reduced function mutations resulted in increased grain set per spikelet, grain number, and yield. The authors stated that the level of *GNI-1* expression had decreased during the domestication of wheat, thus enabling grain number increases.

***Rht-1*** with reduced height alleles, *Rht-B1b* and *Rht-D1b*, are the basis of a well-known story (e.g. Hedden, 2003) in which a gain-of-function mutation arose from a premature stop codon which effectively removed the DELLA domain of GRAS proteins on chromosomes 4B and 4D, respectively, resulting in a semi-dwarf phenotype (Peng *et al.*, 1999) together with increased grain number per unit area. For this reason, semi-dwarf alleles of *Rht-B1b* and *Rht-D1b* have been deployed in most of the world’s irrigated spring wheat and winter wheat varieties (and in many rainfed areas as well). The hypothesis suggested for the molecular function of the semi-dwarfing alleles *Rht-D1b* and *Rht-B1b* was that a methionine that occurs after the N-terminus premature stop codon acted as a new site for translational initiation so that a new shorter peptide carrying the growth-inhibiting GRAS domain was no longer subject to regulation by GA. However, the presence of these N-terminal truncated RHT-1 proteins had not been confirmed until recently (Van De Velde *et al.*, 2021). This recent study also showed that re-initiation occurs in the stem to reduce height, but not in the aleurone layer of wheat seeds where this type of GA insensitivity would cause excessive seed dormancy. These very specific changes in the developmental profile required for a desirable agronomic outcome go some way to illustrate why many dwarfing genes do not produce as desirable an agronomic phenotype as *Rht-1*.

***TaAPO-A1*** is the wheat orthologue of Aberrant Panicle Organization in rice (Kuzay *et al.*, 2019, 2022; Muqaddasi *et al.*, 2019) on chromosome 7A in wheat. A mutation in the F-box domain defines two common alleles in modern global bread wheat which are strongly associated with spikelet number. Association with grain yield is weak, which probably reflects the physiological constraints on achieving increased grain number via spikelet number.

Wang *et al.* (2017) identified several transcription factors that appeared to affect spikelet and floret number. When a subset of three transcription factors was overexpressed as single transgenes, strong correlations were found between their expression and spikelet number, floret number, and seed number per spike, suggesting that the expression of single transcription factor genes can influence spike development

**Box 2. Phenology genes modifying grain number**

There is an increasing body of evidence suggesting a role for phenology genes in increasing grain number and yield. The small number of genes shown to have a reasonably direct and consistent positive effect on grain number in wheat and also in rice, maize, and barley reveals something about the genetic control of grain set. Grain set is a highly plastic process, and the maximization of grain set is tightly linked with the adaptation of a crop to the environment in which it is growing. That is why the very first breeding activity undertaken when a new crop is incorporated in a region is adjusting the time to anthesis and, when optimized, other traits can then be improved. This is because optimizing the time to anthesis is critical for maximizing reproductive output (Fig. B1). In the WDs, this is represented by the boxes at the bottom of the diagram: phenology, total crop cycle, time of anthesis, and terminal spikelet to anthesis.

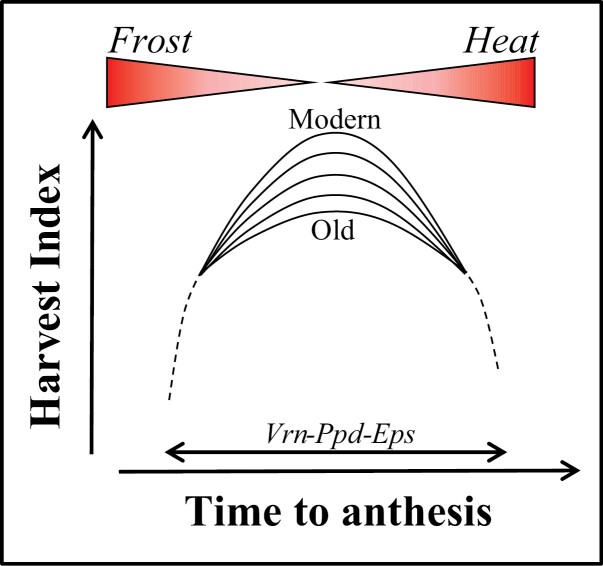

Fig. B1. Schematic representation of changes in harvest index associated with time to anthesis for a range of cultivars varying constitutively in their partitioning patterns (e.g. from very old to modern cultivars). The scheme illustrates why adaptation via adjusting time to anthesis is critical to maximize reproductive output: earlier or later than optimum times to anthesis penalize sink strength through damage from frost or heat, respectively. The most important genetic factors controlling time to anthesis are categorized as earliness *per se*, vernalization, and photoperiod sensitivity (*Eps*, *Vrn*, and *Ppd*).

The genetics of phenology in wheat are relatively well understood. The genes controlling winter/spring growth habit (*Vrn-1*, expressed more highly in the young spike after vernalization, Li *et al.*, 2018) and photoperiod response (*Ppd-1*, expressed highly when spikelets are differentiated, Li *et al.*, 2018), which are responsible for coarse-tuning time to anthesis, are well described elsewhere (Snape *et al.*, 2001; Cockram *et al.*, 2007; Bentley *et al.*, 2013; Bloomfield *et al.*, 2018; Hyles *et al.*, 2020) and are often completely fixed in breeders’ gene pools that target a specific environment. Changing these environmental responses indeed affects grain number, but in the wrong direction when elite germplasm is already well adapted. However, QTLs with smaller effects on phenology are always present, even within the most mature breeding programmes. These are collectively recognized as *Eps*, earliness per *se*, genes and are critical for fine-tuning time to anthesis as well as for the duration of particular subphases composing time to anthesis (Appendino and Slafer, 2003; Lewis *et al.*, 2008; Alvarez *et al.*, 2016; Ochagavía *et al.*, 2018; Basavaraddi *et al.*, 2021b, c). This suggests that disruptive selection generates different combinations of phenology alleles to maintain and enhance genetic gain. The importance of phenology in the WD presentation is reflected by the compartmentation of the WDs into phenology subphases: stem extension; booting; anthesis; watery ripe; and maturity (Figs 4, 5, 8, 9). Five links in this review (Links 1, 13, 14, 29, and 34) relate to wires connected to these phenology phases.

Examples of relationships between genes and phenology are provided by QTLs on chromosomes 1D and 3A which were first identified as heading date QTLs in several UK and CIMMYT wheat populations (Griffiths *et al.*, 2009; Sukumaran *et al.*, 2016). Near isogenic lines (NILs) were developed, validating the heading date QTLs and resulting in a significant grain number effect in both cases. More detailed physiological dissection (Ochagavía *et al.*, 2017) demonstrated that this grain number increase was due to increased floret survival. The 1D QTL effect was shown to be controlled by *Ta-ELF3* (Alvarez *et al.*, 2016; Zikhali *et al.*, 2016). For the QTL on chromosome 3A, fine-mapping of the phenology and grain number effect suggested that the most likely candidate was *FT2* (Martinez *et al.*, 2021). *FT2* has been further implicated in grain number in transgenic studies (Shaw *et al.*, 2019) and diagnostic haplotypes proposed (Glenn *et al.*, 2021).

**Table 1.** A summary of genetic effects contributing to variation within the various links (trait×trait) of the WD

Trait–trait	Reference	Main finding in relation to WD	Gene(s)	Chromosome	Validation
Grain set×yield	Sakuma *et al.* (2019)	This gene is an HD-Zip I transcription factor which is expressed most abundantly in the most distal floret primordia where the authors suggest it inhibits growth. Reduced function mutants produce more fertile florets.	*GN1-1A*	2A	Tilling, RNAi
	Peng *et al.* (1999)	*Rht-D1b* dwarfing alleles increase grain number.	*Rht-1*	4B and 4D	NIL, transgenic
	Muqaddasi *et al.* (2019); Kuzay *et al.* (2019, 2022)	Both studies showed that the wheat homologue of the rice gene *Aberrant panicle organization 1* (*APO-1*) controls spikelet number in bread wheat.	*TaAPO-A1/WAPO1*	7A	GWAS
	Griffiths *et al.* (2009)	Meta QTL analysis identified a number of Earliness *per se* QTLs, some of which were later shown to also effect grain number.	QTL	Multiple	Meta QTL
	Sukumaran *et al.* (2016)	This work showed that a major Earliness *per se* gene (*EPS-D1*) identified in the UK was also important in CIMMYT germplasm and these phenology effects were later shown to influence grain number.	*Eps-D1*/ *TaELF3D1*	1D	GWAS
	Alvarez *et al.* (2016); Zikhali *et al.* (2016)	These studies showed that *EPS-D1* corresponded to *ELF3* which is a component of the circadian clock.	*EpsD1*/*TaELF3D* and *EpsAm1*	1D and 1A	NIL, fine map
	Martinez *et al.* (2021)	A yield QTL identified in the Avalon×Cadenza population and validated using NILs was mapped at higher genetic resolution and shown to co-locate with heading date effects for which the best gene candidate was *TaFT2*.	*TaFTA2* (candidate)	3A	NIL, fine map
	Shaw *et al.* (2019)	This work supports an important role for *FT2* in grain number determination, with transgenic manipulation of the gene producing changes in various fertility traits.	*TaFT2*	Group 3	Transgenic
	Glenn *et al.* (2022)	A single amino acid substitution was associated with significant increases in grain number per spike in bread and pasta wheat.	*TaFT2*	3A	NIL and population
Grain size×yield	Snape *et al.* (2007)	The identification of a major QTL for grain size underwent intensive subsequent study.	QTL	6A	NIL (later studies)
	Farre *et al.* (2016)	Grain size QTL validated in the Avalon×Cadenza NIL library.	NIL effects	2A, 5A, 6A	NIL
	Huang *et al.* (2015)	A robust set of QTLs identified in Chinese germplasm for grain size were validated in NILs showing that marker-assisted selection for this trait is effective and relatively facile.	QTL	2D, 4B, 5A	NIL
	Guan *et al.* (2019)	As above.	QTL	4A	NIL
	Calderini *et al.* (2021)	Overexpression of an α-expansin in early developing wheat seeds leads to a significant increase in grain size without a negative effect on grain number, resulting in a yield boost under field conditions.	Expansin		Transgenic
	Simmonds *et al.* (2014)	Probably the most intensively studied grain size QTL in wheat.	*TaGW2-A1*	6A	NIL, tilling
Crop growth×duration of TS–An	Borras-Gelonch *et al.* (2012)	Independent genetic control of phenology stages shows that, if it is beneficial to allocate more time to the TS–An phase, the genetic variation to do this is available.	QTL	Multiple QTLs	None
	Guo *et al.* (2016)	Detailed glasshouse study using GWAS to map genes controlling phenology phases.	MTA	Multiple	None
Internode length and dry matter partitioning×spike partition index	Peng *et al.* (1999)	*Rht-1* was cloned and shown to be a DELLA domain growth inhibitor. The wild-type protein (full length) is degraded when GA levels increase so that growth is no longer supressed. This sensitivity is removed in the semi-dwarf plants so that growth repression (stem extension) is constitutively expressed so more assimilate can be directed towards the spike.	*Rht-1*	Homoeologous loci on group 4 chromosomes. Semi-dwarfing alleles reported on 4B and 4D.	NIL, transgenic
Spike partition×spike growth	Abeledo *et al.* (2019)	This study shows that when the trouble is taken to collect these phenotypes, QTLs are detected, in this case for spike dry weight at anthesis.	QTL		None
	Rebetzke *et al.* (2011)	*Rht13* is an NBS-LRR class of gene with the *Rht13b* allele autoactivated to reduce height to a similar extent to *Rht1* semi-dwarfing alleles and, like those alleles, can also act to increase grain yield through grain number.	*Rht13*	7B	NIL, tilling
	Cui *et al.* (2011); Zhang *et al.* (2017); Abeledo *et al.* (2019)	Physiological studies show that even when optimal height is achieved in wheat, further fine-tuning is possible by reducing the length of specific internodes. These studies show that QTLs can be identified for internode specific length variation.	QTL	Multiple QTLs	NIL
	Miralles and Slafer (1995); Flintham *et al.* (1997)	Both studies use NILs in very diverse backgrounds to show that genetic variation at th*e Rht1* locus reduces height, increases harvest index, and increases grain yield by increasing grain number.	*Rht-1*	See above	NIL
	Tang *et al.* (2021)	This work shows that *Rht18* can confer the same advantages as Rht-1 without some of the negative effects such as reduced coleoptile length. Ford *et al.*, (2018) showed that *Rht18* is associated with increased expression of *GA2ox-A9*, which encodes a GA-inactivating enzyme that reduces bioactive GA levels.	*Rht18* (major gene not cloned)		
Fruiting efficiency×grain set	Pretini *et al.* (2020b)	In a doubled haploid population (Baguette 19×BIOINTA 2002) increasing alleles for a fertile floret efficiency QTL acted through increased grain set.	QTL	5A	F2
Floret development×fruiting efficiency	Prieto *et al.* (2020)	This shows how Earliness *per se* genes alter seed number by influencing floret survival, but the direction of effect is temperature dependent, so maximizing grain number via phenology is a genetic balancing act that depends on environmental factors.	*ELF3* and *FT2* (candidates)	1D and 3A	NIL
Time of anthesis×fruiting efficiency	Giunta *et al.* (2018)	Fruiting efficiency QTLs were identified and their relationship to phenology dissected.	QTL	Multiple	None
Duration of TS–An×floret development	Basavaraddi *et al.* (2021b, c)	Both QTLs affected the spike fertility by altering the rate of floret development and mortality.	QTL	2B and 7D	NIL
Spike vascular architecture×floret development	Sang *et al.* (2010)	No publications could be found for QTLs for variation in spike vasculature, but Sang *et al*. did find QTLs for small and large vascular bundles of the peduncle.	QTL	Multiple	None
Ovary size×grain weight potential	Brinton *et al.* (2017)	A robust QTL controlling a 7% increase in grain weight shown to be due to increased pericarp cell length.	QTL	5A	NIL
Grain weight potential×grain weight realization	Chapman *et al.* (2021)	Independent EMS-induced mutants expressing delayed senescence and increased grain yield by extended grain fill were shown to have undergone amino acid changes in subdomain 4 of the NAC domain of NAM1.	*NAM-A1* and *NAM-D1*	6A and 6D	Major gene, tilling, NIL
Endosperm cell number/maximum water content of grain×grain weight potential	Bednarek *et al.* (2012)	Cell number has been shown to underlie single gene effects on grain size, as in this study.	*TaGW2*	Group 6	RNAi
Spike hormones–grain weight potential	Guo *et al.* (2022)	*TaCYP78A5* participates in auxin synthesis pathway and promotes auxin accumulation and cell wall remodelling in ovary. Localized overexpression of *TaCYP78A5* in ovary results in delayed flowering and prolonged proliferation of maternal integument cells, which promote grain enlargement.	*TaCYP78A5*		Transgenic
Sink strength×source strength	Mason *et al.* (2013)	Source and sink strength are captured in many studies as biomass components at harvest. Here a Halberd×Karl92 population was used to show the co-localization of grain yield and biomass QTL.	QTL	3B	None
Protein/starch synbthesis×grain weight realization	Uauy *et al.* (2006)	Grain yield and protein concentration are negatively correlated but yield can be increased without decreasing protein if genes controlling grain protein difference (GPD) are exploited. In this work it was shown that NAM-B1 variants can increase grain protein without decreasing grain yield.	*NAM-B1/Gpc-B1*	6B	NIL, transgenic
Duration of grain fill×grain weight realization	Chapman *et al.* (2021)	Independent EMS-induced mutants expressing delayed senescence and increased grain yield by extended grain fill were shown to have undergone amino acid changes in subdomain 4 of the NAC domain of NAM1.	NAM-A1 and NAM-D1	6A and 6D	Major gene, tilling, NIL
Source strength×grain weight realization	Chapman *et al.* (2021)	Source strength was increased through delayed senescence.	NAM-A1 and NAM-D1	6A and 6D	Major gene, tilling, NIL
Source strength×protein/starch synthesis	Uauy *et al.* (2006)	This study showed that a NAC transcription factor was a major regulator of senescence in wheat.	*NAM-B1*	6B	NIL, fine map, transgenic
	Chapman *et al.* (2021)	Independent EMS-induced mutants expressing delayed senescence and increased grain yield by extended grain fill were shown to have undergone amino acid changes in subdomain 4 of the NAC domain of *NAM1*.	*NAM-A1* and *NAM-D1*	6A and 6D	Major gene, tilling, NIL
Carbohydrate storage and remobilization×starch synthesis	Yang *et al.* (2007)	Several studies had identified QTLs for stem soluble carbohydrate reserves and, as in this case, the remobilization efficiency of those reserves (RESWC) and effect on yield.	QTL	1A, 3B, 7A	None
Protein synthesis×starch synthesis	Numerous	The strong negative correlation between yield and protein content means that almost all yield QTLs correspond to an opposite effect for protein.			

For well-studied traits with high heritability, studies in which causative genes have been identified are prioritized. When most evidence is at the level of QTL studies or genome-wide association studies (GWAS), the support of independent validation using unrelated populations and/or NILs is used to help prioritize studies for inclusion. In several cases, the only level of genetic evidence (if any) is based on QTL studies without further validation. Priority is also given to studies in which the wider deployment of beneficial alleles is most likely to lead to progress in modern breeding programmes. In cases where no publications were found in spite of best efforts, this is stated. In all but a few cases, these studies are based on yield measured in field experiments with wheat grown in replicated plots

### Main traits and relationships determining sink strength before anthesis

#### Crop growth and partitioning of assimilates between organs

It has been hypothesized that increasing the relative duration of the TS–An phase would improve grain number by allowing more time for spike biomass accumulation during this critical period, and thereby increasing floret growth at anthesis (Link 1; Fig. 4). This strategy may also lead to increased grain number for a particular level of floret formation by deploying more spike biosynthetic capacity to promote grain set [thereby increasing fruiting efficiency (FE): number of grains per unit spike dry weight at anthesis) (Slafer *et al.*, 2001; Miralles and Slafer, 2007; Guo and Schnurbusch, 2015; Pérez-Gianmarco *et al.*, 2019).

This strategy has also been suggested from modelling exercises (e.g. Sylvester-Bradley *et al.*, 2012). In some field comparisons of varying genotypes, extended duration of this critical phase was related to yield, albeit only weakly (e.g. Gonzalez *et al.*, 2011a; Basavaraddi *et al.*, 2021d; see also Wang *et al.*, 2017), while in others this trait was beneficial in favourable environments (Botwright Acuña *et al.*, 2019). The diversity of results may be due to the cultivars differing not only in the duration of this phase but also in many other traits that affect grain number and yield.

Variability in the duration of this phase independently (at least partially) of total duration of time to anthesis has been reported among elite material (Whitechurch *et al.*, 2007; García *et al.*, 2011; Borras-Gelonch *et al.*, 2012; Botwright Acuña *et al.*, 2019; Basavaraddi *et al.*, 2021a), and seems to be selectable in breeding (Botwright Acuña *et al.*, 2019). Furthermore, specific QTLs for the duration of this phase have been identified (e.g. Borras-Gelonch *et al.*, 2012). An important exercise will be to analyse the location of genes underlying these QTLs where the expression levels correlate with the duration of spike development (Wang *et al.*, 2017).

Improved photosynthesis during the stem elongation phase (see Murchie *et al.*, 2023) would result in improved growth of the juvenile spikes per unit land area (Link 2; Fig. 4). Maintaining spike partitioning could be achieved, for example, by avoiding tiller mortality and/or by increasing growth of the individual spikes. Proof of concept experiments have been conducted where the provision of resources affecting crop growth has been manipulated. For instance, the yield response to CO_2_ enrichment in FACE (free-air CO_2_ enrichment) experiments is consistently related more to improvements in grain number than to improvements in average grain weight (e.g. Ainsworth and Long, 2005; Sun *et al.*, 2009; Fitzgerald *et al.*, 2016), which is compatible with the idea that the yield advantage arises from the increased crop growth during the TS–An critical period for grain number determination. Another proof of concept can be found in a study combining an extensive range of nitrogen (N) doses and timings of application. Yield increases were always related to increases in grain number and spike dry weight at anthesis, but the response was similar for crops fertilized from early in the season or from the onset of stem elongation, implying that growth during TS–An rather than simply total biomass at anthesis or maturity was the critical aspect determining yield responses to fertilization (Fischer, 1993; Fischer *et al.*, 1993; Dreccer *et al.*, 2000). Proof of concept is also provided by genotypic effects. It was demonstrated in a segregating population that QTLs for crop growth rate during the critical period of TS–An could be detected and that the genotypic differences in this rate explained most of those in spike growth and spike dry weight at anthesis (Abeledo *et al.*, 2019). Similar results had also been reported earlier for a different population grown in two contrasting environments (García *et al.*, 2014). Finally, in another proof of concept research, genotypes possessing erect canopies that increase RUE and biomass, discussed in the companion paper (Murchie *et al.*, 2023), increase yield through increasing grain number (Richards *et al.*, 2019).

Increased grain growth requires increased allocation of assimilates to the spikes at anthesis (spike partitioning index; SPI: the ratio between spike and aboveground dry mass at anthesis: Link 3; Fig. 4) as was achieved by the gibberellin (GA)-insensitive semi-dwarf alleles (Miralles and Slafer, 1995; Flintham *et al.*, 1997; Peng *et al.*, 1999) and independent QTLs such as those described by Abeledo *et al.* (2019). Assimilates partitioned to the spike determine the proportion of floret primordia that survive to become competent florets at anthesis (Fischer, 1985; Miralles *et al.*, 1998) and later grains. There is a negative association between spike and stem partitioning of assimilates because rapid growth of stem and spike coincides during stem elongation (Figs 1, 2), especially from booting to anthesis. Beyond GA-insensitive *Rht-B1b* and *Rht-D1b* genes, other dwarfing genes such as *Rht13* (Rebetzke *et al.*, 2011) or *Rht18* (Tang *et al.*, 2021) have been shown to increase grain yield, and others such as *Rht8* may also increase yield but only under particular environmental conditions (Kowalski *et al.*, 2016). These results support the general hypothesis that reductions in stem growth can favour increased metabolite flow to spikes, florets, and grains.

As plant height is a critical determinant of yield (Richards, 1992; Miralles and Slafer, 1995; Flintham *et al.*, 1997; Lopes *et al.*, 2018), it would have been optimized and fixed in wheat traditional growing regions, and further increases in spike growth would require the identification of sources of variation favouring partitioning of assimilates towards the juvenile spike independent of further reductions in plant height. Alternatively, reductions would be limited to small, specific stem internodes to favour spike growth (Link 4; Fig. 4) as proposed by Rivera-Amado *et al*. (2019), when the elite materials are taller than the lower limit of optimal plant height (Richards, 1992; Miralles and Slafer, 1995). Such alternatives were recently demonstrated with CIMMYT elite wheats that showed variation for an enhanced SPI related to increased spike growth before anthesis (Rivera-Amado *et al*., 2019). In this collection of 26 spring wheats, stem–internode lengths (for the peduncle, internode 2, and internode 3) were measured as proxies for stem–internode dry matter at GS65 + 7 d and strong negative associations between lengths for internodes 2 and 3 and spike growth (which occurs simultaneously) were observed. Sierra-Gonzalez *et al.* (2021) similarly observed in a panel of 150 CIMMYT spring wheat genotypes that stem–internode 3 length was negatively associated with spike partitioning index and grain number per unit area, supporting reducing the length of internode 3 as a strategy to increase spike partitioning and grains per unit area whilst maintaining lamina partitioning and lamina growth (Link 5; Fig. 4). Several studies have identified QTLs which control height by a disproportionate reduction in the length of specific internodes (e.g. Cui *et al.*, 2011; Zhang *et al.*, 2017). However, these studies did not include the measurement of SPI or grain yield.

Spike photosynthesis also plays an important role as a source of photoassimilates during grain filling, not only under drought, but also under optimal agronomical conditions (Araus *et al.*, 1993; Tambussi *et al.*, 2005, 2007; Maydup *et al.*, 2010; Sanchez-Bragado *et al.*, 2014a; Link 6; Fig. 5). Therefore, plant development profiles that favour spike growth in the pre-anthesis phase will also increase post-anthesis source strength through enhanced spike photosynthesis, helping to maintain optimum source–sink dynamics in modern cultivars.

All hypotheses and models incorporating transfer of source photoassimilates to the sink to achieve high sink growth activities must consider not only the rates of photosynthate generated and the diverse ways it is used in source materials, including storage, but also the efficiency of transport to sink tissue. There is now a general understanding of, for example, sucrose transport from leaves and stems to sink tissue that has been gained over the past 10 years in monocots, especially through molecular genetic studies on rice. The many transporters involved, their locations, and the energy requirements to move sugars in and out of vacuoles, through the phloem and into sink cells, will have a large effect on the overall ability of sink cells, tissues, and organs to grow and contribute to grain yield. Such systems may be rate limiting for yield (Braun *et al.*, 2014; White *et al.*, 2016). This hypothesis is supported by research on transgenic wheat plants expressing high levels of the AVP1 H^+^-PPase transporter gene from Arabidopsis (Regmi *et al.*, 2020). The transgenes in these plants, constitutively expressed under the control of a maize ubiquitin promoter in the spring wheat cultivar Bobwhite L. stimulated augmented the presence of H^+^-PPase in the collection phloem and higher rates of carbon transfer into developing grains and higher yields, with higher numbers of grains per spike, in both greenhouse and field assays. The plants also showed enhanced carbon partitioning between shoots and roots. Further evidence of the importance of transfer of source photoassimilates to the sink is provided by studies where overexpression of the barley sucrose transporter, HvSUT1, under the control of an endosperm-specific promoter, enhanced sucrose flux into wheat grains. This resulted in elevated grain biomass and grain Zn and Fe content of transgenic winter wheat lines in field micro-plots (Saalbach *et al.*, 2014) and grain protein content in greenhouse and field conditions compared with the wild-type cv. Certo (Weichert *et al.*, 2010). Interestingly, CO_2_ fertilization negated these phenotypic responses in the transgenic lines, pointing to upstream factors regulating sucrose import into, or maternal transport within, developing grains (Weichert *et al.*, 2017).

Transport of amino acids, metal ions, and other molecules is also vital for high yields. Transport of amino acids is dependent on supplies and storage of N compounds that late in the plant life cycle can be limiting. In addition, sucrose and nitrate are signalling molecules (e.g. Krouk *et al.*, 2010; Tognetti *et al.*, 2013; Lastdrager *et al.*, 2014), and so the efficiency of these signalling effects may also contribute to the efficiencies of source to sink transfer of assimilates and essential metabolites. Finally, spike growth may be enhanced by manipulating the trehalose-6-phosphate (T6P) signalling system (Paul *et al.*, 2020). Genetic and chemical intervention approaches have been used to modify the T6P pathway and improve performance of wheat (Paul *et al.*, 2018), and this aspect of sugar utilization can contribute to enhanced partitioning of sucrose into spikes.

#### Floret development and fruiting efficiency

Yield increases due to environmental effects, such as N fertilization, are related to changes in spike dry weight at anthesis (e.g. Fischer, 1985, 1993; Prystupa *et al.*, 2004; Slafer *et al.*, 2022) as a consequence of more floret primordia becoming fertile florets (Ferrante *et al.*, 2020), supporting the proposed hypothesis that floret developmental rates reflect the resources allocated to the growing juvenile spikes (Ferrante *et al.*, 2013a; Dreccer *et al.*, 2014). Indeed, floret primordia mortality seems to be triggered by the trophic relationships associated with the initiation of the active growth of the juvenile spikes (González *et al.*, 2011b; Ferrante *et al.*, 2013b). Also, the relationship between number of grains set (or the number of fertile florets at anthesis) and spike dry weight at anthesis (Link 7, Fig. 5) is maintained during genetic improvement (e.g. Brooking and Kirby, 1981; Fischer and Stockman, 1980; Acreche *et al.*, 2008). Much of the genetic gain obtained to date is due to the improvement of the growth of juvenile spikes, in large part due to the introgression of the *Rht* genes as described above (e.g. Siddique *et al.*, 1989; Slafer and Andrade, 1993). However, not all genotypes translate resources transferred to the growing juvenile spike into grains with the same efficiency (Slafer *et al.*, 2015, 2022). Fruiting efficiency is a key trait which reflects this (Link 8, Fig. 5). Fruiting efficiency (i.e. the efficiency for using dry matter allocated to the juvenile spike before anthesis to set a certain number of grains, determined as the number of grains per unit spike dry weight at anthesis; Slafer *et al.*, 2015), is a measure of the outcome of processes related to floret development pre-anthesis (Fig. 5), as well as grain abortion post-anthesis (Fig. 8). There is genetic variability in fruiting efficiency among modern wheat cultivars that correlates well with grains per unit area (González *et al.*, 2011a; Elía *et al.*, 2016; González-Navarro *et al.*, 2016; Rivera-Amado *et al.*, 2019; Slafer *et al.*, 2022) with QTLs and identified MTAs (e.g. Gerard *et al.*, 2019).

Improvements in fruiting efficiency may arise from an accelerated rate of floret development and improved partitioning of spike assimilates between the main body of the spike and its developing florets (Link 9, Fig. 5). In fact, both might be linked because increasing the allocation of resources to spikes increases the rate of floret development (Ferrante *et al.*, 2013a; Dreccer *et al.*, 2014). Genetic variation in the rate of floret development in the absence of variation in spike growth seems possible (Ferrante *et al.*, 2020) where a difference in floret development is due to improved intra-spike assimilate partitioning favouring florets over the structural tissues of the spike (Foulkes *et al.*, 2011; Slafer *et al.*, 2015; Rivera-Amado *et al.*, 2019). Indeed, variation in intra-spike partitioning, with a concomitant increase in biomass occurring in developing florets instead of structural components of the spike (rachis, glumes, and so on), has been shown not only between old and modern cultivars (Slafer and Andrade, 1993) but also within modern germplasm (Abbate *et al.*, 1998; García *et al.*, 2014). However, there are uncertainties about the potential for manipulating this partitioning effect within the juvenile spike as the physiology of this partitioning has not been studied in great detail (Fischer, 2007) and assessments of potential drawbacks require more data and further analysis (Foulkes and Reynolds, 2015). There is also evidence that genetic variation in fruiting efficiency is influenced by levels of spike hormones (see later). Several QTLs (although not yet validated) for fruiting efficiency have been reported (e.g. Giunta *et al.*, 2018), and should be useful for breeding because the trait is heritable, exhibits transgressive segregation (Martino *et al.*, 2015), and responds to selection (Pedro *et al.*, 2012; Alonso *et al.*, 2018). During the anthesis to watery ripe period, influences on fruiting efficiency may also result from grain abortion (i.e. failure of fertile florets to set grains; see later).

There may exist a trade-off between fruiting efficiency and spike dry matter (Link 10, Fig. 5), as reported in independent studies (e.g. Dreccer *et al.*, 2009; Lázaro and Abbate, 2012), particularly when differences in grains per m^2^ are small (Slafer *et al.*, 2022). However, many cultivars having both high fruiting efficiency and high spike weight at anthesis have been identified (Bustos *et al.*, 2013; García *et al.*, 2014; Elía *et al.*, 2016; Ferrante *et al.*, 2017). This suggests there is no feedback regulation between these traits, and it is possible (and likely) to achieve an increase in one with no compensating responses from the other. Nonetheless, in breeding for improved fruiting efficiency, breeders should be alert to any trade-off with spike dry weight at anthesis and should select against it in the progeny being selected.

Floret initiation during the phase from the onset of stem elongation to booting is only marginally responsive to spike growth. Therefore, genotypes differing strongly in the allocation of resources to spike growth and then spike fertility do not differ in the maximum number of viable floret primordia just prior to booting (e.g. NILs for *Rht* genes have clear differences in spike fertility but similar maximum numbers of florets initiated; Miralles *et al.*, 1998). Thus, environmental factors affecting growth before booting barely affect the number of primordia initiated (Ferrante *et al.*, 2013a). During the following period, from booting to anthesis, floret development is highly responsive to spike growth, or vice versa (Link 11, Fig. 5). Most proximal florets normally develop to produce a fertile floret, while distal floret primordia always die before becoming a fertile floret (e.g. Ferrante *et al.*, 2020), probably because they have a rather delayed onset of development (Backhaus *et al.*, 2022). Labile florets in intermediate positions of the spikelets either progress towards producing fertile florets or die, which is critical in determination of overall spike fertility. The potential of each of these intermediate floret primordia to progress through normal development and become a fertile floret, or alternatively abort, depends on the influx of resources to the growing juvenile spike. This has been demonstrated by altering the dynamics of spike growth using environmental interventions (Ferrante *et al.*, 2013a) as well as studying genetic variants (Miralles *et al.*, 1998; González *et al.*, 2011b) including NILs for cloned QTLs (Prieto *et al.*, 2020).

It has been postulated that, during the evolution of the wild grass ancestors, natural selection favoured the production of a very large number of floret primordia, regardless of the conditions, whose development is dependent on the subsequent availability of resources (Sadras and Slafer, 2012). Genetic strategies to test and exploit this hypothesis include reducing the duration of the TS to the initiation of booting phase while increasing the duration of booting to anthesis. Although several studies on the timing and duration of these phases have been published (e.g. Guo *et al.*, 2018), limited evidence for the effects of the specific reduction of the duration of this phase on spike growth and floret development is available.

There may be a trade-off between floret development and grain abortion (Link 12, Fig. 5). Ovary size at anthesis is associated with both floret survival (pre-anthesis) and grain abortion (post-anthesis), providing a connection between these two traits (see later). Thus, assimilates available to distal florets may play a critical role in regulating both floret survival and grain setting (Ferrante *et al.*, 2020).

Timing of anthesis directly affects sink strength (Link 13, Fig. 5) because it influences the period of floret development and so the ambient temperature experienced by those florets. Suboptimal temperatures immediately before anthesis reduce fruiting efficiency, when anthesis is earlier (low-temperature damage to floral organs) or later (heat effects on floret fertility) than optimum for each particular environment. High temperatures >31 °C) can lead to sterility through floret abortion and/or reduction of pollen tube development, and increases of pollen mortality in wheat (Prasad and Djanaguiraman, 2014). A one pot temperature transfer study showed the ‘double dip’ effect of high temperature at phases that might correspond to meiosis and microsporogenesis (Barber *et al.*, 2017). There are also effects of low temperatures, with meiosis at the early booting stage identified as the most sensitive period (Thakur *et al.*, 2010; Ji *et al.*, 2017). In the UK and France, cold and wet weather during floral development in the wheat variety ‘Moulin’ caused significant sterility and a reduction in grain yield of >70% (Law, 1999) due to low temperatures at meiosis. It was suggested that Moulin might carry specific alleles from diverse sources that made it more sensitive to cold weather during meiosis. The meiotic recombination gene *Dmc1* on wheat chromosome 5D has been identified as a candidate for the maintenance of normal chromosome synapsis and crossover at low and possibly high temperatures (Draeger *et al.*, 2020). Meiosis is preceded by a long pre-meiotic cell cycle where many epigenetic events take place. This phase may be very sensitive to temperature and other stresses that can lead to floret abortion. An examination of the portfolio of genes highly expressed during these phases revealed that those which confer stress tolerance are especially active during the double ridge phase (Li *et al.*, 2018), indicating that spike development is inherently stressful, particularly due to poor supply of nutrients.

Given that anthesis date is usually the first trait optimized in breeding programmes, any lengthening of the duration of the critical phase for grain number determination (TS–An) would further indirectly increase the number of florets that develop normally through increasing the growth accumulated in that phase (see above; Link 1, Fig. 5). There may also be a direct effect: if the onset of floret development is not proportionally delayed, there would be additional time for the development of individual florets, allowing some labile florets to develop into fertile florets instead of stopping their development and dying (Link 14, Fig. 5). As floret initiation is far less responsive to genetic and environmental factors than floret mortality (Sadras and Slafer, 2012) during the period of booting to anthesis (when floret mortality takes place), extending the duration of the phase provides more time for floret development, thereby increasing the number of florets that develop normally (Fig. 6).

Extension of the floret development period does not affect the progress of the most robust primordia (e.g. florets most proximal to the rachis) but increases the chance of labile floret survival (e.g. the fourth or even fifth floret position in central spikelets and florets 2–3 of more basal and apical spikelets). These labile florets would stop developing in other circumstances (contributing to floret mortality) but, provided with an extended period of development, can progress to fertile florets instead of dying before anthesis (González-Navarro *et al.*, 2015; Prieto *et al.*, 2018; Perez-Gianmarco *et al.*, 2019).

In summary, the likely effect of a longer duration of stem elongation would function more through floret survival than simply floret initiation (e.g. González *et al.*, 2005; Guo *et al.*, 2016; Fig. 6), thereby principally affecting fruiting efficiency; and would be more relevant during the phase from booting to anthesis rather than TS to booting. Empirical support for this was reported by Wang *et al.* (2017) in demonstrating that expression levels of single genes can lead to more florets and a longer duration of spike development and by González-Navarro *et al.* (2015) who demonstrated that fruiting efficiency showed a positive trend with the duration of stem elongation. A boundary function suggested that the length of this phase may impose a threshold for fruiting efficiency and grain number, and that maximum fruiting efficiency may require both generation of many florets and a relatively long stem elongation phase, as illustrated by manipulating daylength in the field to make the period from TS to anthesis longer or shorter, affecting floret development (Gonzalez *et al.*, 2003, 2005; Serrago *et al.*, 2008).

#### Hormones and vascular architecture

There is increasing evidence that variation in fruiting efficiency is regulated by plant growth regulators during the rapid spike growth phase from booting to anthesis (Link 15, Fig. 5). Auxin and cytokinin (CK) are key regulators during meristem formation, and their interactions regulate meristem differentiation and function. CKs stimulate cell division and nucleic acid metabolism, and are known to be associated with grain number in wheat (Sakakihara, 2006). Higher CK signalling levels occur before the double ridge phase which helps retain meristem activity, while high auxin activity is up-regulated from the double ridge stage, most probably contributing to the generation of new axillary meristems (Li *et al.*, 2018). However, there is a negative correlation between auxin concentration and the number of fertile florets at the abortion stage. Adding exogenous 6-benzylaminopurine (6-BA, a synthetic CK) during the abortion phase increased the CK level, reduced the auxin level, decreased the number of floret abortions, and increased spike dry weight (Li *et al.*, 2019). Thus, increasing CK levels in spikes during the floret abortion phase results in increased grain number, for example, by decreasing cytokinin oxidase activity that catalyses the degradation of CK (Bartrina *et al.*, 2011; Zhang *et al.*, 2011). CK levels are regulated by a balance between biosynthesis [e.g. isopentenyl pyrophosphate transferase (IPT)] and degradation [e.g. cytokinin oxidase/dehydrogenase, CKX)] enzymes. The grain sink strength of the spike meristem could therefore be enhanced by altering CK homeostasis through the up-regulation or down-regulation of these enzymes, respectively, to coordinate growth and floret fertility (Li *et al.*, 2019). While there has been speculation on a possible trade-off between fruiting efficiency and spike dry weight at anthesis (see above; Link 10, Fig. 5), manipulating spike hormones may offer breeders one avenue for simultaneously raising both.

Hanif and Langer (1972) showed that floret fertility is dependent on spike vascular architecture (Link 16, Fig. 5). Therefore, improved knowledge of vascular bundle development and connectivity is required to better understand floret survival in wheat. There is evidence for branching of the main vascular bundles in the spike rachis (Kirby and Rymer, 1974; Whingwiri *et al.*, 1981; Wolde *et al.*, 2019), but it is not yet clear how the assimilates are allocated to each of the branch units—the spikelets. It has been suggested that florets closer to the rachis node (i.e. the basal three florets in the spikelets) are directly supplied by the principal vascular bundles of the rachilla, while the distal florets lack a direct connection to the vascular bundle (Hanif and Langer, 1972) and therefore might not have an equal chance of accessing assimilates from the source. Wolde *et al.* (2019) postulated that the vascular structure in the wheat spikelet might relate to the wide conduits assigned at the base of the spikelet feeding the narrower conduits of the distal florets. In summary, if florets are formed normally with a good potential for viability, the flow of assimilate may be too limiting to support all rapidly growing florets because of features of the spike vascular architecture.

The number and arrangement of each spikelet on the spike are under strong hormonal control (McSteen, 2009; Poursarebani *et al.*, 2015; Dixon *et al.*, 2018). However, relatively little is known on the role of plant hormones in regulating the development of the spike vascular architecture (Link 17, Fig. 5), especially the main conducting elements (i.e. the sieve tube elements) and their architectural configurations in the spikelet/floret. There is evidence that the PIN-FORMED1 (*PIN1*) efflux carrier concentrates auxin into local maxima in the epidermis, which position incipient leaf or floral primordia in angiosperms. In *Brachypodium distachyon*, transgenes for the duplicate *PIN1* clade members *PIN1a* and *PIN1b* were shown to stimulate the transport of auxin from these maxima into internal tissues along emergent paths that pattern leaf and stem vasculature (O’Connor *et al.*, 2014). Therefore, the *PIN1* genes may have a role in determining the paths that pattern spike vasculature. Genes controlling wheat spike architecture and spikelet arrangement have already been reported (Boden *et al.*, 2015; Dixon *et al.*, 2018; Wolde *et al.*, 2019), but to date no genes determining spike vascular architecture have been identified. In summary, the elucidation of spike vascular architecture and its genetic regulation is at an early stage and requires more study.

#### Ovary size and grain weight potential

As discussed above (Link 12, Fig. 5), a possible mechanism for increasing fruiting efficiency would be reduction of the resource threshold required for floret development (Slafer *et al.*, 2015). This would permit a larger proportion of initiated florets to survive, but these would all be reduced in size (Dreccer *et al.*, 2009). This would not be ideal as the improvements in fruiting efficiency would come at the cost of compensatory reductions in grain weight (Link 18, Fig. 5). Thus, this avenue for improving fruiting efficiency would probably be inefficient in improving yield (Ferrante *et al.*, 2015).

The connection between ovary size and final grain weight is based on the fact that potential grain weight is related to the size of the ovary (Calderini *et al.*, 2001; Hasan *et al.*, 2011; Xie *et al.*, 2015; Reale *et al.*, 2017). This relationship has been demonstrated regardless of the source of variation, that could be either genotypic (Calderini and Reynolds, 2000; Yu *et al.*, 2015; Simmonds *et al.*, 2016) or environmental (Wardlaw, 1994; Calderini *et al.*, 1999; Ugarte *et al.*, 2007). Because the ovary wall becomes the pericarp of the grain, the size of the ovary determines the upper limit for grain growth (i.e. grain weight potential; Link 19, Fig. 5). Thus, the final size of the grains would appear to be regulated by maternal tissues, because grain weight potential is a critical determinant of final grain weight (Link 20, Fig. 5).

Final grain weight is the result of a balance between potential size of the grains and the capacity of the crop to realize this potential during the effective period of grain filling. As the capacity of the source to fill the grains seems to be in excess of the demands of the growing grains (see also below), grains normally grow as much as they can; that is, they mostly they do not respond to manipulations of source strength per growing grain from the onset of the effective period of grain filling. This significantly reduces their plasticity (compared with that of grain number; Sadras, 2007; Slafer *et al.*, 2014) and is a major cause for the much higher heritability of final grain weight compared with any other yield component (Sadras and Slafer, 2012). The final grain weight then depends on the capacity of the grains themselves to grow, an attribute thats is genotypically determined by the potential size of the grains and environmentally regulated by factors affecting the capacity of the grains to fulfil that potential (e.g. high temperatures during the effective period of grain filling reduce grain weight by directly affecting their capacity to grow, in addition to any effects they may also have on leaf senescence). The potential grain weight is determined prior to the onset of the effective grain filling (Calderini *et al.*, 2001; Fahy *et al.*, 2018; Xie *et al.*, 2015) by the size of the ovary determined during booting to anthesis (Link 19, Fig. 5), and the number of endosperm cells and maximum water content determined during anthesis to watery ripe (see below).

These latter two inter-related traits (number of endosperm cells and maximum water content) are likely to be pre-determined by the ovary size (Link 21, Fig. 5). There is evidence showing a positive relationship of grain weight potential to either ovary size (Singh and Jenner, 1982; Calderini *et al.*, 1999; Hasan *et al.*, 2011), number of endosperm cells (Brocklehurst, 1977; Gleadow *et al.*, 1982), or maximum water content (Saini and Westgate, 1999; Pepler *et al.*, 2006; Hasan *et al.*, 2011). For all three traits, the independent variables reflect the sink capacity of each single grain: the ovary wall becomes the pericarp of the grain, the endosperm cells are the units where starch will be stored, and the maximum water content provides a reference to the volume of the grain. Furthermore, when analysed together, ovary size, water accumulation, and grain dimensions are probably controlled by the same QTLs (Xie *et al.*, 2015).

Ovary size at anthesis represents a possible predictor for grain setting (Guo *et al.*, 2016) and is naturally related to the allocation of resources to the spike (Link 22, Fig. 5). In the field, spike dry weight was positively associated with ovary size for 30 European winter wheat genotypes (Guo *et al.*, 2016), probably due to a longer duration of pre-anthesis phases (see above). Ovary size has been reported to have high heritability (Komaki and Tsunewaki, 1981; Guo *et al.*, 2015), and QTLs for grain size, validated for their effects on grain yield, have been identified (Simmonds *et al.*, 2014).

## WDs from anthesis to maturity

As discussed above and in a previous paper (Reynolds *et al.*, 2022), it is mainly during the pre-anthesis period when yield potential is determined. During the post-anthesis period, potential yield is finalized and actual yield is achieved from this potential through the process of grain weight realization.

‘Grain filling’ comprises two subphases that contribute differently to yield (Fig. 7). The first occurs during ~7–10 d (depending on temperature) from anthesis to the stage of watery ripe grain, when potential yield is finally established, thereby determining sink strength during the effective period of grain filling. In the embryo, this period is marked by the establishment of the new genotype following fertilization, during which many epigenetic events establish the potential gene expression profiles in the embryo and at later developmental stages.

During this first period, both the final number of grains and their final potential size are established through (i) the level of abortion determined by the proportion of fertile florets setting grains that grow normally afterwards, and (ii) the final volume of the grains to be subsequently filled with assimilates (Fig. 7). During this period, overall development is very active, with multiplication of endosperm cells, but there is virtually no growth of the grains (in terms of dry matter gain). This period is commonly known as the ‘lag phase’, referring to the delay before initiation of dry matter gain. The second subphase is significantly longer (~25–50 d, depending on temperature) and known as the effective period of grain filling (Fig. 7). The completion of the effective grain-filling phase defines the physiological maturity of the crop.

As in the pre-anthesis WD, the period from anthesis to maturity is divided into two WDs to provide a detailed description of key traits. The first focuses on traits determining sink strength from anthesis to watery ripe (Fig. 8). The second focuses on the effective grain-filling period from watery ripe to maturity (Fig. 9).

### The relevance of grain weight to determination of harvest index and yield (major Link B, Fig. 8)

#### Relevance of grain size in wheat

Final grain size is one of the two major yield components (Slafer *et al.*, 2014) and is therefore a critical factor to consider when attempting to improve yield, particularly as it has a relatively high heritability (significantly higher than the number of grains set by the crop; e.g. Egli, 1981; Kuchel *et al.*, 2007; Sadras and Slafer, 2012), and several genetic factors have already been identified that could be exploited (see below).

Conversely, grain size is a rather conservative trait exhibiting far less variation than grain number (e.g. Peltonen-Sainio *et al.*, 2007; Sadras, 2007) and there are evolutionary reasons (also exploited in breeding) why grain size is less plastic than grain number (Sadras and Slafer, 2012). As such, the expected magnitude of yield improvement that might be envisaged through selecting for larger grains would probably be modest. An indirect proof of concept for this is that wheat breeding has more frequently made yield advances by improving grain number (despite the expected difficulties with the low heritability of grain number, as already described in Link A; although exceptions can be identified (e.g. Aisawi *et al.*, 2015)). In addition, breeders have usually sought greater homogeneity in the weights of individual grains in seed lots (Finch-Savage and Bassel, 2016), because when grains undergo industrial processes such as milling, high variability in weight adversely affects the nutritional and processing quality of the end-products.

A factor that partly explains the low plasticity of grain size is the lack of source restrictions (considering together post-anthesis photosynthesis and remobilization of pre-anthesis stored reserves) to fill the grains (see also Links 30 and 31, Fig. 9). However, as past breeding has consistently exploited this avenue through increasing grain number, current elite material might be exhibiting an incipient co-limitation from both source and sink strengths (e.g. Shearman *et al.*, 2005; Acreche and Slafer, 2009; Aisawi *et al.*, 2015), although, even in these cases of incipient ‘co-limitation’, yield is far more sink limited than source limited during grain filling. Thus, the relevance of grain size may increase in future breeding efforts to raise yield potential. While this restriction in variability for grain size is linked directly to effective grain growth, final grain size also depends on the potential size of the grains. Therefore, achieving positive impacts on yield by improving grain size through increasing grain weight potential may be possible (see details and references in Link 20). However, this would only be effective if potential trade-offs between the generation of grain number and potential grain weight are recognized and avoided (see Links 18 and 22), for instance, through the selection of germplasm with higher grain weight potential (and normally higher final grain size) achieved without sacrificing grain number (see discussion on this trade-off in Slafer *et al.*, 2015). The fact that empirical evidence shows that it is possible, in some lines, to combine high grain number with larger grains (e.g. Bustos *et al.*, 2013; Calderini *et al.*, 2021) is encouraging.

#### Genetic variation for grain size in wheat

There is a rich body of literature describing the genetic architecture of this trait and the molecular genetics underpinning wheat grain development, probably due to the high heritability of grain size. It is generally the case that QTLs for grain size are not accompanied by QTLs with positive effects for yield. Indeed, alleles responsible for increasing grain size usually co-locate with QTLs for decreased grain number per unit area, reflecting the physiological trade-off already described (e.g. Zhai *et al.*, 2018). Due to the unbalanced nature of this trade-off, in the direction of grain number, there is quite often a significant yield penalty that accompanies increasing grain size. Therefore, the challenge for future yield improvement is to devise strategies that will enhance the scope for potential grain weight to respond to availability of assimilates without unduly enhancing the risk of incomplete grain filling.

It will be important for such work to be underpinned by genetic analysis, and it is encouraging that, for a range of different wheat crosses, several QTLs and MTAs controlling grain yield have been discovered that influence individual grain weight without pleiotropic effects on grain number (Snape *et al.*, 2007; Calderini *et al.*, 2021). A detailed review of the genetics and development of the wheat grain is given in Brinton *et al.* (2018). Here we focus on variants for grain size that appear to minimize the trade-off and so show promise for the improvement of grain yield within current breeding programmes that target high yield potential environments. This also means that these effects need to have been validated beyond their initial description as QTLs, MTAs, or induced variants (Table 1). It should be emphasized that grain size is important beyond grain yield. The manipulation of grain size also influences early plant establishment, specific weight, milling efficiency, and market preference. Our criteria for further description should not be taken to mean that the genetic effects not described in detail here are unimportant.

Robust and NIL-validated grain size effects have been described on wheat chromosomes 2A (Farre *et al.*, 2016), 2D (Huang *et al.*, 2015), 4A (Guan *et al.*, 2019), 4B (Huang *et al.*, 2015), 5A (Farre *et al.*, 2016; Huang *et al.*, 2015), and 6A (Simmonds *et al.*, 2014; Farre *et al.*, 2016). Of these, it is only the QTL on chromosome 6A which has been shown to have a consistent beneficial effect on grain yield. This QTL was first identified in multiple UK elite winter wheat populations (Snape *et al.*, 2007; Ma *et al.*, 2015) and then validated (Simmonds *et al.*, 2014; Farre *et al.*, 2016). Candidate genes for this QTL have been studied in some detail, in particular the wheat orthologue of *GW2* from rice. Su *et al.* (2011) and Zhang *et al.* (2013) both identified significant associations of *TaGW2* alleles with grain size. Simmonds *et al.* (2016) used tilling populations to identify a G to A transition in the splice acceptor site of exon 5 of *TaGW2-A1* which leads to mis-splicing of the mRNA transcript and larger grains. These results increase our knowledge of the genetics of grain size, but some uncertainty remains as to whether *TaGW2* and these associated alleles really underlie the 6A QTL for grain size and yield effects. The first two studies show association of opposite alleles with the increasing effect, and the rate of recombination on chromosome 6A is so low that most of the genes mapped on it are present in very few haplotypes. This would suggest that *TaGW2* is a negative regulator of grain size in wheat, similar to the function in rice, but is probably not the same as the yield-increasing effect identified by Simmonds *et al.* (2014). It is most likely that the expansive 6A haplotypes carry multiple genes that influence different yield components (Sukumaran *et al.*, 2018; Brinton *et al.*, 2020).

### Main traits and relationships determining sink strength after anthesis

#### Grain abortion

Grain number depends principally on the number of fertile florets that are pollinated but also on the proportion of grains which abort before starting their active growth. The latter process takes place in the ‘lag phase’ of ~7–10 d after anthesis (the period from then to watery ripe grains). After anthesis and pollination have occurred and before grains start to grow, there is a critical process of grain set responsible for the proportion of fertile florets effectively becoming growing grains. This is when the maternal and paternal sets of chromosomes undergo epigenetic changes and embryo development is initiated. Although in elite germplasm, including modern cultivars, most fertile florets successfully develop a grain, a variable number of grains abort before they start growing (abortion ranging in modern cultivars under non-stressed field conditions from virtually zero to ~40%; Siddique *et al.*, 1989; González *et al.*, 2003; Elía *et al.*, 2016; Guo *et al.*, 2016). This process seems to be sensitive to environmental and genetic stresses (e.g. Savin and Slafer, 1991; Hays *et al.*, 2007). For example, high temperatures (Prasad and Djanaguiraman, 2014) and lack of N availability (Ferrante *et al.*, 2013a) can increase grain abortion. Guo *et al.* (2016) demonstrated a large genetic variation in grain set in both field and glasshouse conditions and across all spikelet positions studied, although the trait also displayed moderate levels of heritability. More recently it was established that grain set was a relevant component of genetic variation in fruiting efficiency (Pretini *et al.*, 2020a). Thus, grain abortion is a component of fruiting efficiency (Link 23, Fig. 8), and improvements in the latter could be achieved through reducing grain abortion.

One possible avenue to reduce grain abortion would be to select against florets of very small size, as small florets probably produce abortive grains (Link 24, Fig. 8). Fertile florets vary greatly in size, depending on their position in the spikelet as well as the position of the spikelet in the spike and the chronological order of the spike-bearing tiller. Allowing more floret primordia to become fertile florets without proportional increases in availability of resources for floret growth would increase the proportion of fertile florets that are small. This occurs normally with the most distal florets becoming fertile. Empirical data show that the size of the ovaries at anthesis could be used as a predictor for the probability of grain setting (Guo *et al.*, 2015). Guo *et al.* (2016), comparing contrasting genotypes for the size of the ovaries of the fourth floret from the rachis (a distal floret), reported that smaller ovaries led to increasing probabilities of grain abortion. This reinforces the hypothesis that for fruiting efficiency to be a relevant trait for yield improvement, it should not be achieved at the expense of having smaller florets but instead by increasing intra-spike partitioning favouring floret growth. Empirical evidence supports the fact that genotypic differences in fruiting efficiency could be independent of concomitant differences in ovary size (Elia *et al.*, 2016).

Grain abortion may also be affected by spike hormones (Link 25, Fig. 8). It has been observed that excessive ethylene production results in wheat grain abortion under high temperature stress (Hays *et al.*, 2007), suggesting that grain accumulation of ethylene may be a trait to target. Similarly, a negative association was observed at high temperatures in the field between spike dry weight at anthesis and ethylene production in a genome-wide association study population, and the genetic basis underlying this trait was suggested (Valluru *et al.*, 2017). High ethylene levels can reduce grain yields in maize by accelerating embryo and grain abortion, thereby reducing sink size (Shi *et al.*, 2015). In addition, there is a clear indication that accumulation of abscisic acid (ABA) in developing grains of maize can result in grain abortion (Wang *et al.*, 2002). In addition, CKs during the grain abortion phase have been identified as playing a potentially relevant role in increasing sink strength (Zalewski *et al.*, 2010; Li *et al.*, 2019). In summary, pinpointing the plant hormone signals underlying grain abortion and their genetic control in wheat may allow the development of genotypes with a less conservative strategy for determination of grain number.

It also seems likely that grain abortion may be influenced by the spike vascular architecture (Link 26, Fig. 8), especially for the distal florets which are too small to avoid abortion after pollination (see above; Link 23, Fig. 8), but more needs to be known about this. The vascular system of the spike is sufficiently different from the pattern encountered in vegetative nodes to warrant unique study (O’Brien *et al.*, 1985). The basal three florets in the spikelets appear to be directly supplied by the principal vascular bundles of the rachilla, while the distal florets may lack a direct connection to the vascular bundle (Hanif and Langer, 1972; O’Brien *et al.*, 1985). Thus, mechanical modifications to improve the vascular connectivity of the bundles supplying the distal florets might increase assimilate translocation and reduce grain abortion. However, little is presently known about how sieve tube elements in the bundles are distributed in wheat spikelets and how a single sieve tube bifurcates at each junction of the branches. Therefore, further study of the phloem anatomy and structure in wheat spikelets to develop a mechanistic working model using the appropriate genetic material is important to exploit this potential avenue to reducing grain abortion (Wolde and Schnurbusch, 2019).

#### Grain weight potential

Grain weight potential, together with the number of grains set, is the final component determining sink strength during grain filling. Grain weight potential is initially determined through attributes related to the size of the ovary (see above) and complemented by the number of endosperm cells that are produced in each of the grains (Link 27, Fig. 8). Endosperm formation is initiated by very rapid DNA synthesis without cell division to produce a coenocytic bag. The number of rounds of DNA replication influences the number of endosperm cells and hence grain weight potential. The endosperm consists of cells with widely differing shapes and sizes, with large numbers of small, regularly shaped cells beneath the aleurone layer and small tubes of large, isodiametric cells in the central parts of the tissue (MacMasters *et al.*, 1971). Despite this variety of cell types, the final endosperm cell number is related to final grain weight, as it is in these cells that starch (by far the largest component of grain dry matter; Stone and Savin, 1999; Shewry, 2009) is stored. Endosperm DNA content and cell number are both positively associated with mature grain weight among a range of genotypes. However, not all of the variation in grain weight can be attributed to variation in cell number because of differences in mean dry weight per endosperm cell (Chojecki *et al.*, 1986). Genetic variation exists for the rates of increase of DNA and cell number which are related to final grain weight (Bennett *et al.*, 1975).

The switch from mitotic division to endoreduplication is associated with decreases in mitotic CDKs (cyclin-dependent kinases; protein kinases regulating the cell cycle) and increases in S phase CDKs (Sabelli and Larkins, 2009). If endoreduplication boosts the number of cells to drive rapid carbohydrate and protein synthesis, then the numbers of rounds of endoreduplication is relevant to define potential grain size. Zeatin is high in early endosperm formation when cell division and the mitotic index are high (1–3 d), while indole acetic acid (IAA) is boosted later when endoreduplication occurs. Endosperm filling has been thought to be determined by the rate of transport of sugars (and N) into endosperms, and the energy available for biosynthesis. This appears to be rate limiting per endosperm because if florets are removed then the remaining endosperm/grains become larger (Calderini and Reynolds, 2000). However, Calderini *et al.* (2021) recently illustrated (see fig. 1 in their paper) that the magnitude of endosperm cell division might be a response to the size of the ovary, because the number of endosperm cells could be linked to the size of the ovary (Hasan *et al.*, 2011; Brinton and Uauy, 2019; Kino *et al.*, 2020). When florets are removed (Calderini and Reynolds, 2000), the remaining florets produce a larger ovary that would produce, in turn, a greater number of endosperm cells, and the rate of transport of sugars (and nitrogen) into endosperms would be sink controlled. Therefore, the priority and rate at which carbohydrates and amino acids enter the developing grain from anthesis to watery ripe through vascular connections would strongly affect grain weight potential. Therefore, spike vascular architecture may be a key target not only to reduce grain abortion (see above) but also to increase grain weight (Link 28, Fig. 8). This suggests that a better understanding of the vascular bundle development, connectivity, and metabolite transport would be important for understanding the determination of potential grain weight.

Timing of anthesis determines the temperatures to which the crop is exposed at the time the ovaries are growing (the few days immediately before anthesis; see above) as well as during the ‘lag phase’ from anthesis to watery ripe, when grain endosperm cells are developing. Thus, the date of anthesis may affect the number of endosperm cells produced because it reflects the thermal regime to which the cells were exposed (Link 29, Fig. 8). It has been shown that temperature regimes immediately before anthesis may affect not only abortion of floret primordia and then grains but also the size of the carpels in those florets that attain the stage of becoming fertile. This is because of the effects on ovary size and thus grain weight potential (see above). Empirical evidence suggests that potential grain weight may be influenced by the temperatures occurring immediately before anthesis (Calderini *et al.*, 1999; Ugarte *et al.*, 2007), which agrees with the proposed model indicating that the period immediately before anthesis is critical for defining grain weight potential (Calderini and Reynolds, 2000; Calderrini *et al.*, 2001). Furthermore, Nicolas *et al.* (1984) showed that reductions in grain weight due to exposure to high temperature were due, in turn, to the reduction in the number of endosperm cells. High temperatures during the lag phase may affect both the number and size of endosperm cells (Kaur *et al.*, 2011).

Finally, when grain weight potential is fixed, the sink strength of the crop during the effective period of grain filling (from watery ripe to physiological maturity) is established. This level of sink strength established by the crop may influence the levels of canopy photosynthesis during grain filling (Link 30, Fig. 9). Although in theory yield potential of wheat would be determined by photosynthesis over the entire growing season, it may be that photosynthesis influences yield increases only during periods of crop growth that are source limited (see conclusions in Reynolds *et al.*, 2005; and the paper introducing the concept of the WD; Reynolds *et al.*, 2022). There have been reports, among contrasting genotypes, associating post-anthesis canopy (including spike) photosynthesis with yield when comparing modern/semi-dwarf and old/tall genotypes (e.g. Calderini *et al.*, 1997; Miralles and Slafer; 1997; Reynolds *et al.*, 2005, 2001). Intuitively it may be interpreted that the improved photosynthetic rates resulted in the yield improvements. However, evidence suggests otherwise. Improved sink strength results in increases in photosynthesis and RUE during the effective period of grain filling, and leaf, spike, and canopy photosynthesis was down-regulated from watery ripe to maturity when there is insufficient sink strength (e.g. Acreche and Slafer, 2009; Serrago *et al.*, 2013). This conclusion, from experiments manipulating source–sink balances, is in line with the evidence coming from increasing sink strength genetically (e.g. Calderini *et al.*, 1997; Miralles and Slafer, 1997), and was further proven when strategic crosses done in a realistic breeding context demonstrated that increasing sink strength boosts RUE (Reynolds *et al.*, 2017).

#### Grain weight realization

Grain weight realization is related to the rate and duration of grain growth. Genetic variation exists for both processes, and positive relationships are reported between final grain weight and each of these two components (most commonly the rate of grain filling; e.g. Calderini and Reynolds, 2000; Charmet *et al.*, 2005; Xie *et al.*, 2015). However, as the availability of assimilates (actual photosynthesis plus available reserves) seems to be in excess of the sink capacity to store them during the effective period of grain filling (e.g. Borras *et al.*, 2004; Reynolds *et al.*, 2005; Cartelle *et al*., 2006; Serrago *et al.*, 2013; Borrill *et al.*, 2015; as discussed in the previous paper; see fig. 1 in Reynolds *et al.*, 2022), the relationship between grain filling and final grain weight is less relevant than that between potential and actual grain weight. Clearly, grain weight realization will always be tightly linked to final grain weight (Link 31, Fig. 9), but this realization depends more on the capacity of the grains to grow than on the availability of assimilates. Thus, despite the high correlation, the link is not considered as having the highest relevance, at least under optimal growing conditions. This is borne out by the fact that considerable amounts of WSCs often remain in the stem when measured at physiological maturity (Foulkes *et al.*, 2002; Serrago *et al.*, 2013). Under a given temperature regime, genetic variation in the duration of grain filling is smaller than in the rate of grain growth (e.g. Sofield *et al.*, 1977; Duguid and Brûlé-Babel, 1994); therefore, only small improvements in final grain weight might be achieved in yield by lengthening the duration of grain filling (Link 32, Fig. 9). The effect of high temperatures on reducing grain weight, however, is more related to duration than rate of grain filling (Sofield *et al.*, 1977; Wiegand and Cuellar, 1981).

There is a well-established relationship between leaf and spike photosynthesis and grain filling in wheat. The relevance of leaves, and particularly the flag leaf, has long been recognized (Evans *et al.*, 1975; Araus and Tapia, 1987). More recently it is widely recognized that post-anthesis spike photosynthesis is also a major contributor of assimilates filling the grains (Tambussi *et al.*, 2007; Maydup *et al.*, 2010). By using different techniques, it has been shown that in general most of the assimilates used to fill the grains effectively come from the actual leaf (and particularly the flag leaf) and spike photosynthesis (Sanchez-Bragado *et al.*, 2014b, 2016) (Link 33, Fig. 9). There is evidence that breeding has improved post-anthesis photosynthesis and RUE (Calderini *et al.*, 1997; Molero and Reynolds, 2020), and recently Molero and Reynolds (2020) demonstrated that spike photosynthesis is a heritable trait. However, some redundancy may exist between particular organs if their photosynthetic contribution is replaced by other sources, including the remobilization of reserves stored in the stems before the onset of grain filling. Further, improving leaf/spike photosynthesis during the effective period of grain filling would probably produce gains in yield, only in cases where grain filling was source limited, a situation that is not common, particularly for potential yielding conditions (see other links in this paper, as well as discussion on spike photosynthesis in the companion paper by Murchie *et al.*, 2023). It follows that in some cases photosynthetic activity during grain filling may be a consequence rather than a cause of increased yield (e.g. Calderini *et al.*, 1997).

Although not the most common situation, there is nevertheless evidence in modern wheat cultivars of some source limitation during grain filling under optimal conditions. In this situation, grain growth is co-limited during grain filling, as sink capacity is limited during early grain filling and source capacity is limited at later stages of grain filling (Acreche and Slafer, 2009). The stay-green trait and extended duration of grain filling (Link 34, Fig. 9) may be associated with yield gains in these situations. Genetic variation for functional stay-green—delayed senescence associated with extended photosynthesis (Thomas and Howarth, 2000)—has been reported in bread wheat (Christopher *et al.*, 2008; Kumar *et al.*, 2010; Gaju *et al.*, 2011; Derkx *et al.*, 2012), and remote sensing has been used to phenotype such genetic variation (Lopes and Reynolds, 2012). Bogard *et al.* (2011) reported that the timing of flag leaf senescence was associated with a QTL for flowering date in a winter wheat Toisondor×CF9107 doubled haploid (DH) population in field experiments in France and the UK. Kumar *et al.* (2010) reported QTLs for a stay-green trait under field conditions in Indian wheat lines using a recombinant inbred population from a cross between ‘Chirya 3’ and ‘Sonalika’, with the QTLs accounting for up to 39% of phenotypic variation. In addition, Christopher *et al.* (2016) identified variation in a SeriM82×Hartog DH population of wheat for specific stay-green traits, combinations of traits, and/or molecular markers related to the traits which were associated with higher yield in both well-watered and water-limited conditions.

As leaves senesce, proteins including Rubisco are degraded and N is remobilized to the grain, resulting in a reduction in photosynthetic capacity (e.g. Feller *et al.*, 2008). Therefore, delayed remobilization of N to grains for protein synthesis is associated with stay-green (Link 35, Fig. 9). Genetic improvement in stay-green traits has been specifically associated with lower post-anthesis N remobilization (Gaju *et al.*, 2011; Derkx *et al.*, 2012; Hawkesford, 2014) and/or increased post-anthesis N uptake (Bogard *et al.*, 2011; Gaju *et al.*, 2014). A transcription factor (NAM-B1) accelerates senescence and increases N remobilization from leaves to grains in wheat (Uauy *et al.*, 2006). Therefore, a better understanding of the mechanisms determining post-anthesis N remobilization and senescence may offer scope to increase grain yield and/or grain protein content in wheat cultivars in these cases of co-limitation of grain growth during grain filling.

#### Stem carbohydrate and its remobilization

The relative contribution of stem carbohydrate remobilization (Link 36, Fig. 9) to grain yield varies widely depending on the environmental conditions and cultivar (Blum, 1998; Foulkes *et al.*, 2002; Ruuska *et al.*, 2006). In general, a reduction in current assimilation under post-anthesis drought will induce greater stem reserve mobilization, and utilization by the grain (Palta *et al.*, 1994; Saint Pierre *et al.*, 2010). However, there is also evidence for significant deposition of stem WSC reserves in grains contributing between 10% and 34% grain yield in the absence of post-anthesis stress in wheat (Gebbing and Schnyder, 1999; Gebbing *et al.*, 1999; Foulkes *et al.*, 2002). The results of Shearman *et al.* (2005) and Saint Pierre *et al.* (2010) confirm the potential importance of stem WSC for grain yield potential even under favourable post-anthesis conditions.

In wheat, stem WSCs are composed mainly of fructans (Ruuska *et al.*, 2006) which are polymers of fructose. The breakdown of fructans supports the growing grains: fructan exohydrolase 1-FEHw3 mapping on chromosome 6B is a useful marker for fructose breakdown (Zhang *et al.*, 2008). Indeed, this gene is a major factor determining genotypic variation in WSC remobilization in wheat (Zhang *et al.*, 2015). Khoshro *et al.* (2014) identified that the wheat cultivar Zagros, possessing enhanced capability for fructan storage and higher mobilization efficiency, had a higher gene expression level of 1-SST, 6-SFT, 1-FEHw3, as well as 6-FEH genes. Expression of 1FEHw3 and 6-FEH increased during carbon remobilization in this cultivar, suggesting that both genes are necessary for an efficient degradation and translocation of stem fructans. The mRNA levels of two fructan synthetic enzymes (1-SST and 6-SFT) in the stem were positively correlated with stem WSC concentrations, while the mRNA levels of enzymes involved in fructan hydrolysis (INV, 1-FEHw3, and 6-FEH) were inversely correlated with WSC concentration. Carbon assimilate availability through stem carbohydrate remobilization should prolong starch synthesis and/or grain growth, therefore enhancing grain weight realization by extending the duration of grain growth.

Grain filling commences 5–10 d after anthesis and continues over the last 25–50 d until the grain ripens. The accumulation of structural proteins (albumin, globulin, and the amphiphilic fraction) occurs up to 25 d after anthesis followed by the storage proteins (gliadin and glutenin fraction) when cell division has ceased (Stone and Nicolas, 1996). The initial accumulation of structural proteins is considered sink regulated, whereas the supply of storage proteins is considered source limited (Martre *et al.*, 2003). The genotype–environment interaction modifies total grain N, through source limitation (Martre *et al.*, 2003). The relationship between grain protein and starch synthesis (Link 37, Fig. 9) is influenced by the concentration of sucrose and glutamine and their ratio in the grain, as affected by the key enzymes sucrose synthase and glutamine synthetase, respectively (Zhou *et al.*, 2006). Since starch and protein deposition in the endosperm of wheat are controlled by separate mechanisms, starch yield and protein yield should therefore be selected as independent traits in breeding.

It has been suggested that grain weight realization might be affected by spike vascular architecture (Link 38; Fig. 9). It is presently unclear whether the provision of sucrose to the grain during the watery ripe to physiological maturity stage is limited by transport system activities and/or spike vascular connections of the grains to the plant, thereby affecting grain weight realization. Under normal conditions, starch synthesis in the developing grain appears not to be restricted by a lack of carbohydrate precursor, as described above. Further studies are required to study the dynamics of grain filling, development of spike vascular tissue, and the rate of dry matter accumulation, in relation to the anatomy and activities of the phloem transport systems. In addition to vascular architecture, it has also been hypothesized that improvements in phloem loading may be useful for enhancing assimilate supply to growing grains (Braun *et al.*, 2014; White *et al.*, 2016), but extensive evidence for this is so far available only from model plants. However, Regmi *et al.* (2020) demonstrated that increasing a specific H^+^-PPase transporter activity via transgenesis led to increased transfer of carbon into wheat grains.

## Concluding remarks

The diagrams presented here and in the companion paper (Murchie *et al.*, 2023) represent—as far as we are aware—the most up-to-date (albeit high level) understanding of the interactions among yield potential traits that has been documented in wheat and based on evidence gathered mostly under agriculturally relevant growing conditions. While presenting the platform organized around WDs as a potential workspace for breeders and other crop scientists, the authors recognize that the details of the WDs and table of genetic information will need updating regularly as knowledge accumulates about trait and gene interactions, and as new traits and their genetic basis are discovered (Reynolds *et al.*, 2022). This is perhaps especially so at the molecular frontier, as such techniques become increasingly applied to tissues from plants grown in realistic crop environments. Such outputs, along with high throughput and precision phenotyping data, measured directly on field-grown plots, and perhaps with the aid of artificial intelligence (AI), will help build new dynamic models of trait and gene interactions in wheat and other field crops.
